# Breaking Supercapacitor Symmetry Enhances Electrochemical Carbon Dioxide Capture

**DOI:** 10.1021/jacs.5c00999

**Published:** 2025-04-29

**Authors:** Zhen Xu, Xinyu Liu, Grace Mapstone, Zeke Coady, Charles Seymour, Selina E. Wiesner, Svetlana Menkin, Alexander C. Forse

**Affiliations:** † Yusuf Hamied Department of Chemistry, University of Cambridge, Cambridge CB2 1EW, U.K.; ‡ Faculty of Chemistry and Pharmacy, Ludwig-Maximilians-Universität München, Munich 81377, Germany

## Abstract

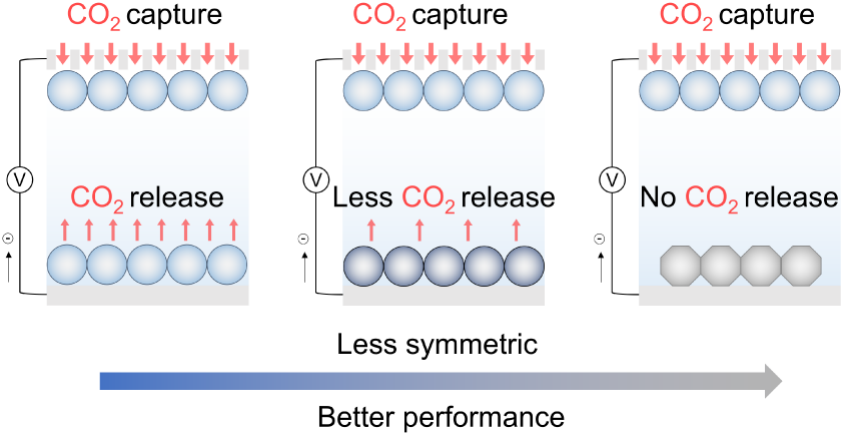

Electrochemical CO_2_ capture using supercapacitors offers an energy-efficient approach for mitigating CO_2_ emissions, but its performance is thought to be hindered by competing CO_2_ capture and release processes at two identical porous carbon electrodes. To address this, we introduce an asymmetric supercapacitor-battery hybrid system with porous carbon and nonporous metallic zinc as the working and counter electrodes, respectively. The CO_2_ capture capacity continuously increases as the charging rate decreases with a maximum capacity of 208 mmol_CO2_ kg^−1^, surpassing that of an analogous symmetric supercapacitor. Our findings suggest that breaking device symmetry enhances CO_2_ uptake in capacitive systems by suppressing competing processes, while the noncapacitive zinc counter electrode simplifies the mechanistic picture of capacitive CO_2_ capture. Extending this approach, we develop asymmetric supercapacitors with two different porous carbon electrodes, demonstrating a 200% increase in CO_2_ capture capacities at low charging rates. In summary, this study pioneers asymmetric systems for electrochemical CO_2_ capture and establishes a general strategy to enhance both understanding and performance.

## Introduction

**Figure 1:**
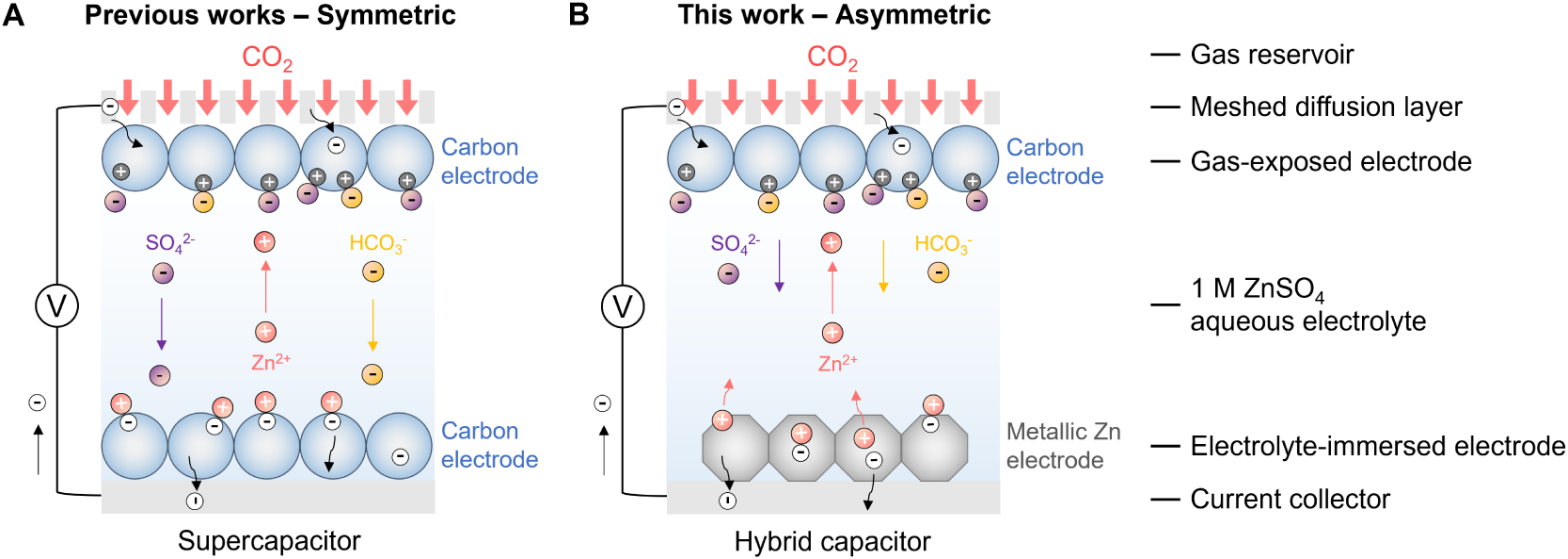
Schematic diagram illustration of electrochemical CO_2_ capture in the symmetric supercapacitor and hybrid capacitor systems during the discharge process of the positively charged gas-exposed carbon electrode. The mechanistic hypothesis (i.e., “ionic liquid-solid” mechanism) for CO_2_ capture and corresponding ion movements during the discharge process of the positively charged gas-exposed carbon electrode in (A) the supercapacitor with a symmetric cell configuration and (B) the hybrid capacitor with an asymmetric cell configuration and a metallic zinc counter electrode. The schematic shows charge storage mechanisms, i.e., electrochemical double-layer capacitive (EDLC) behaviors, on porous carbons possibly involving physical counterion adsorption, co-ion desorption, and counterion-*co*-ion exchange, in comparison to the charge storage behaviors based on the redox couple of Zn^2+^/Zn in nonporous metallic zinc without a large amount of physical ion sorption or exchange. Under the electric field, cations and anions move correspondingly. The formed concentration gradient of CO_2_-derived (bi)carbonate ions could be a driving force to dissolve more CO_2_ into aqueous electrolytes.

Developing sustainable and energy-efficient CO_2_ capture approaches is crucial for realizing net-zero targets.^1^ Electrochemical CO_2_ capture technologies can be powered by renewable electricity as the sole energy source and are emerging as energy-efficient alternatives to traditional CO_2_ capture processes that rely on thermal or pressure swings.^2^ Recent studies have shown that CO_2_ can be captured from simulated flue gases using simple supercapacitors through an effect known as supercapacitive swing adsorption (SSA).^3,4^ The device configuration typically features a symmetric supercapacitor cell consisting of two identical porous activated carbon electrodes and an aqueous electrolyte, with one electrode partially exposed to a CO_2_-containing gas and the other fully immersed in the electrolyte (Figure 1A). While the cell composition is symmetric, the preferential gas contact to one electrode breaks the overall symmetry of the system. Compared with other battery-type electrochemical CO_2_ capture approaches based on electrochemically driven pH swings,^5−7^ redox-active CO_2_-binding molecules^8−11^ and electrochemically mediated amine regeneration,^12−14^ the SSA-based technology exhibits outstanding energy efficiency and stability.^15^ Recent works have optimized electrode materials,^15,16^ electrolyte compositions,^17,18^ and charging protocols^19−21^ for SSA. Even with this progress, there is still a lack of a clear pathway to enhance the relatively low CO_2_ adsorption capacities of SSA (approximately ∼100 mmol CO_2_ per kg of the active mass of the working electrode) owing in part to the incomprehensive mechanistic understanding of SSA.^21^

The “molecular liquid-solid” mechanism proposed that SSA might result from the electro-adsorption of dissolved molecular CO_2_ at the charged surface of carbon electrodes (Figure S1).^22^ A recent study provided support for this “molecular liquid−solid” mechanism on gold electrodes, which was achieved by relating capacitance decreases in the presence of CO_2_ due to its presence in the electrical double layer.^23^ However, this mechanism does not straightforwardly explain our observed dependence of CO_2_ capture and release on the charging polarity of the gas-exposed working electrode when activated carbon electrodes are used,^15^ and the dominant mechanism may ultimately vary depending on the choice of electrode material structure, electrolyte compositions, or experimental conditions.^23^ Therefore, an alternative “ionic liquid-solid” mechanism has been proposed by researchers (Figure S1),^22^ whereby (bi)carbonate electro-sorption in the electrical double layer drives CO_2_ capture (and release) through perturbations to the below equilibria:^22^
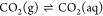
1
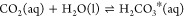
2
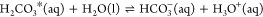
3
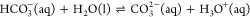
4

In this theory, when the gas-exposed carbon electrode obtains electrons either during the negative charging of a pristine electrode or the discharging of a positively charged electrode, CO_2_-derived (bi)carbonate ions (*e.g.,* HCO_3_^−^) migrate away from the gas-exposed carbon electrode, leading to a local depletion of CO_2_ which drives CO_2_ capture (Figure 1A).^21,24^ Conversely, when the gas-exposed carbon electrode is charged in a positive direction (i.e., when it loses electrons), (bi)carbonate electro-adsorption leads to an accumulation of CO_2_ which drives its release into the gas chamber. Evidence that this mechanism plays a role in SSA was provided by a COMSOL model which allowed (bi)carbonate and CO_2_ concentrations to be monitored during charging, although this model could not rule out the effect of the “molecular liquid-solid” mechanism.^24^

Regardless of the specific dominant mechanism, we recently made a key discovery that when using activated carbon electrodes, SSA operates as an inherently kinetic process in symmetric supercapacitors.^24^ Specifically, we found that when approaching equilibrium conditions through charging the supercapacitor very slowly (i.e., at low currents), or by using very long voltage holds, the net quantity of electrochemical CO_2_ capture was greatly reduced.^24^ We proposed that there are competing CO_2_ capture and release processes at the two electrodes in the supercapacitor, which become most apparent when charging slowly due to the slow mass transport of CO_2_-derived species to/from the electrolyte-immersed counter electrode which is not directly exposed to gas.^15,24^ Namely, CO_2_ capture at the gas-exposed carbon electrode is accompanied by CO_2_ release at the electrolyte-immersed carbon electrode, reducing the overall CO_2_ adsorption capacities of the device.^24^ In pursuit of enhanced CO_2_ capture performance, strategies to reduce these competing processes are needed.

Here we therefore employed nonporous metallic zinc as the electrolyte-immersed counter electrode to break the supercapacitor symmetry (Figure 1B) and to suppress the competing processes in conventional symmetric supercapacitors. Our asymmetric supercapacitor-battery hybrid system (i.e., Zn-ion hybrid capacitor) exhibited an increasing trend in the CO_2_ adsorption capacity with a decreasing applied current density (208 mmol_CO2_ kg^−1^ at 1 mA g^−1^), surpassing that of the symmetric supercapacitor which showed decreased CO_2_ adsorption capacities under slow charging conditions. Our approach introduces an entirely new supercapacitor-battery hybrid system for electrochemical CO_2_ capture and provides insights into the competing processes in symmetric supercapacitors for SSA. Furthermore, this work shows that the concept of asymmetric supercapacitors for SSA can be generally extended using other counter electrode materials (*e.g.,* microporous carbons), proposing a promising general pathway for designing electrochemical CO_2_ capture devices with enhanced performance.

## Results

**Figure 2:**
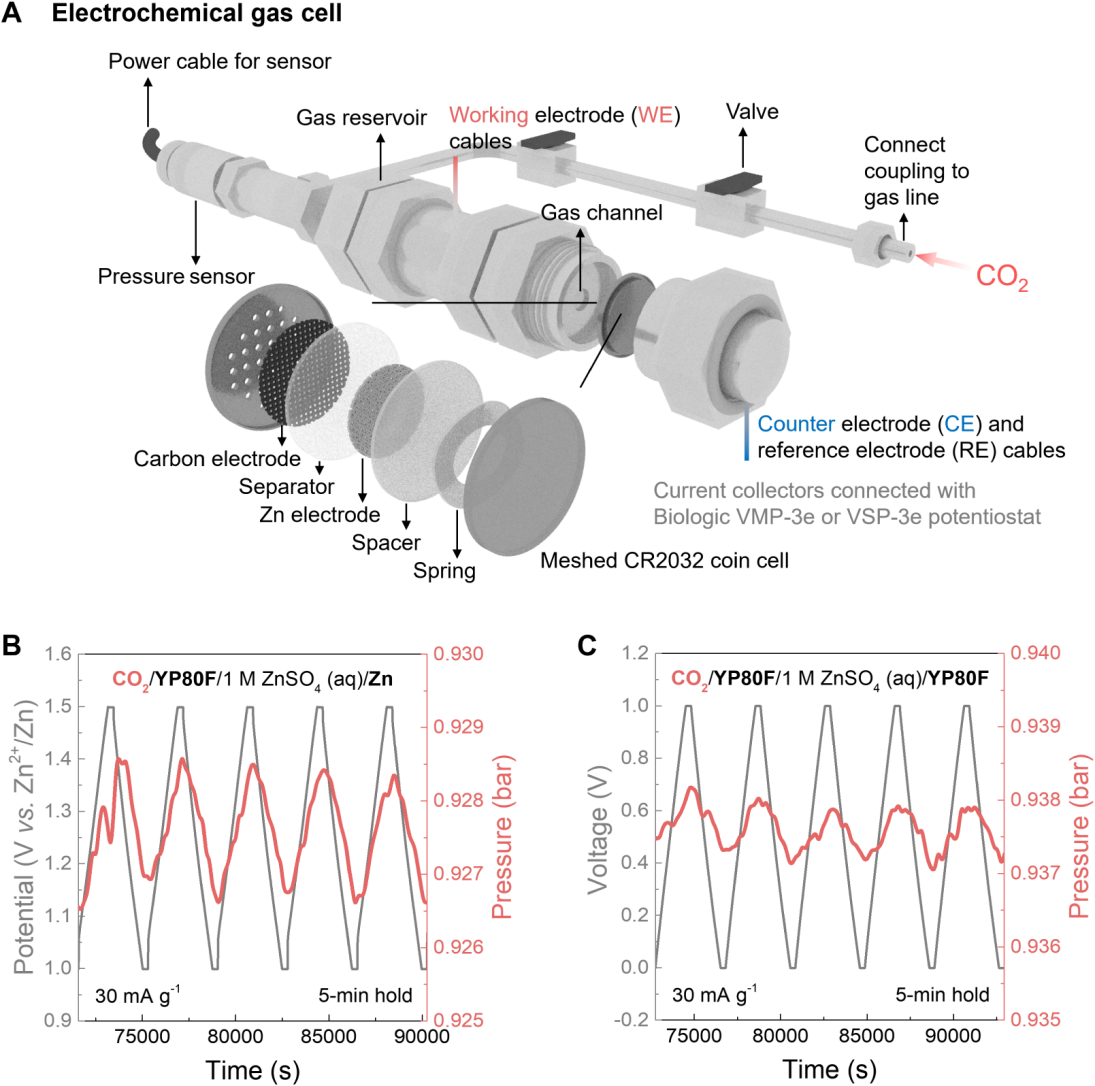
Thermodynamic performance of electrochemical CO_2_ capture by the symmetric supercapacitor and hybrid capacitor systems. (A) Schematic of the custom-made electrochemical gas cell setup that houses a meshed coin cell for electrochemical CO_2_ capture measurements at 303 K. (B) Zoomed galvanostatic charge−discharge (GCD) curves (gray) and smoothed pressure curves (averaged every 100 s, red) of the hybrid capacitor (noted as “CO_2_/YP80F/1 M ZnSO_4_ (aq)/Zn”) under pure CO_2_ at a current density of 30 mA g^−1^ in the positive charging mode, with 5 min voltage/potential holds at the limiting potentials. (C) Zoomed GCD curves (gray) and smoothed pressure curves (averaged every 100 s, red) of the symmetric supercapacitor (noted as “CO_2_/YP80F/1 M ZnSO_4_ (aq)/YP80F”) under CO_2_ at a current density of 30 mA g^−1^ in the positive charging mode, with 5 min voltage/potential holds at the limiting cell voltages. All metrics were normalized by the active mass of the gas-exposed working electrode for both systems.

Using our previously developed electrochemical gas setup (Figures 2A and S1), we studied electrochemical CO_2_ capture by both hybrid capacitors (Figure 2A,B) and symmetric supercapacitors (Figure 2C). Commercial YP80F activated carbon electrodes were used in both systems, with the hybrid capacitor employing a zinc counter electrode in contrast to the symmetric carbon−carbon supercapacitor. For the hybrid capacitor and the symmetric supercapacitor, the cells were labeled as “CO_2_/YP80F/1 M ZnSO_4_ (aq)/Zn” and “CO_2_/YP80F/1 M ZnSO_4_ (aq)/YP80F”, respectively, following their assembly sequences. In both cells, the activated carbon electrode was the CO_2_ gas-exposed electrode in the initial experiments presented here.

Excitingly, the hybrid capacitor reversibly captured and released CO_2_ during discharge and charge, respectively (Figure 2B). When the potential of the activated carbon electrode was made more positive than its open-circuit potential of around 1.0 V vs Zn^2+^/Zn (i.e., “positive charging mode”), a pressure increase was observed, indicating that CO_2_ already dissolved in the electrochemical cell was released (Figures 2B and S2). When the potential of the activated carbon electrode was then decreased back to 1.0 V vs Zn^2+^/Zn, CO_2_ capture was observed *via* a pressure drop. These findings were reproducible across several cycles (Figure 2B). Additionally when the cell was instead “charged” to potentials below its open-circuit potential of around 1.0 V vs Zn^2+^/Zn (i.e., “negative charging mode”), similar CO_2_ capture and release behaviors were observed (Figure S2), indicating that CO_2_ adsorption happened when the gas-exposed carbon electrode obtained electrons for both positive and negative charging modes. The dependence of CO_2_ capture and release on the polarity of the charged state of the carbon electrode is most consistent with the proposed “ionic liquid-solid” mechanism where CO_2_-derived bicarbonate ions are the active species of CO_2_ capture.^15^ This is supported by solid-state nuclear magnetic resonance (NMR) experiments (Figure S3) which demonstrate that bicarbonate ions form when a soaked YP80F electrode is exposed to CO_2_ gas.^25^ However, we cannot rule out a possible contribution from the “molecular liquid-solid” mechanism, given that our solid-state NMR experiments also revealed the presence of molecular CO_2_ inside the carbon pores (Figure S3). Importantly, these initial findings (Figure 2B) show that only a single capacitive electrode is needed to achieve SSA, where the counter electrode serves simply to balance the charge in the electrochemical cell.

The operating potential window of 1.0−1.5 V vs Zn^2+^/Zn was selected for the following measurements of the carbon−zinc hybrid capacitors as side reactions were observed beyond this range. Under cell potentials above 1.5 V vs Zn^2+^/Zn, an irreversible pressure increase was observed, likely due to hydrogen evolution and water oxidation (Figures S2, S4 and S5), while the observed irreversible pressure decrease below 1.0 V vs Zn^2+^/Zn might be derived from parasitic reactions such as corrosion processes that consume CO_2_ (Figures S2, S4 and S5). To compare the CO_2_ adsorption capacity between the hybrid capacitor and symmetric supercapacitor systems, the cell voltage window of the symmetric supercapacitor was selected to be 0.0 − 1.0 V, with the two identical electrodes splitting the overall cell voltage into equal and opposite electrode potentials (i.e., each electrode’s potential changed by 0.5 V) (Figure S6). Therefore, the applied potential on the working electrode in both systems was +0.5 V relative to the open-circuit value (Figures S6 and S7). Additionally, a 5 min voltage/potential hold was applied to minimize the effects of slow CO_2_ sorption kinetics and cell polarization (from no voltage hold) as well as side reactions (from long voltage holds) (Figures S8 and S9).

We next compared the thermodynamic CO_2_ capture performance of both hybrid and symmetric cells using these voltage/potential parameters and a fixed current density of 30 mA g^−1^. The hybrid capacitor (Figure 2B) and the symmetric supercapacitor (Figure 2C) displayed qualitatively similar CO_2_ sorption behaviors. In both cases, CO_2_ was adsorbed when the gas-exposed carbon electrodes obtained electrons and desorbed when electrons were released, consistent with our previous observations in 1 M NaCl (aq) and 1 M Na_2_SO_4_ (aq) electrolytes for symmetric supercapacitors.^21^ The activated carbon in the hybrid capacitor showed an electrochemical capacitance of 99 F g^−1^ (i.e., the ability to store charge at a given voltage), similar to that of the symmetric supercapacitor (i.e., 102 F g^−1^). However, the CO_2_ adsorption capacity of the hybrid capacitor (i.e., 89 mmol_CO2_ kg^−1^) was significantly higher, compared to 41 mmol_CO2_ kg^−1^ for the symmetric supercapacitor, as seen by the magnitudes of the pressure oscillations (Figure 2B vs 2C). We hypothesize that the enhanced CO_2_ adsorption capacity for the hybrid capacitor arises from the lack of competing CO_2_ capture/release processes at the counter electrode,^24^ i.e., the nonporous metallic zinc counter electrode can balance the charge stored at the carbon electrode (through zinc plating and stripping based on the redox couple of Zn^2+^/Zn with a certain potential)^26^ without capturing or releasing CO_2_ (without significant sorption of CO_2_ or CO_2_-derived (bi)carbonate ions).

The corresponding electrical energy consumption for CO_2_ capture using the hybrid capacitor was decreased to 51 kJ mol_CO2_^−1^ because of the enhanced CO_2_ adsorption capacity, which was much lower than 126 kJ mol_CO2_^−1^ required by the symmetric supercapacitor. Future decreases in energy consumption can be achieved through strategies such as materials optimization for enhanced CO_2_ adsorption capacity and cell engineering for reduced internal resistance. Furthermore, the hybrid capacitor potentially offered a higher energy density (i.e., 16 Wh kg^−1^) than the symmetric supercapacitor (i.e., 7 Wh kg^−1^), when normalized by the working electrode mass. However, we cannot rule out the role of the metallic zinc and inactive materials on the device energy density in practical energy storage applications. The selective participation of CO_2_-derived bicarbonate ions in the charge compensation processes also needs consideration. The estimated ionic charge storage capacities derived from CO_2_ capture were lower than the electrochemical charge storage capacities of the working electrode (Table S1), indicating the possible competition in ion migration and storage between bicarbonate ions and other electrolyte species (e.g., SO_4_^−^ anions).^27^

Notably, experiments on a symmetric zinc−zinc cell configuration under identical conditions showed no measurable CO_2_ capture/release behavior during charging/discharging cycles (Figure S10), confirming that the carbon electrode is the active component for CO_2_ capture/release in our system. We further note that the horizontal baselines of the pressure curves represented CO_2_ diffusion equilibration, negligible side reactions, and no system leakage (Figure 2B,C), which were also supported by the leak test and rest measurements before the charge−discharge processes (Figure S10). The use of meshed coin cells allowed good contact between the gas-exposed working electrode and the gas reservoir, thus ensuring reproducible cell assembly and avoiding high cell resistances (Figure S11).^15^

In terms of stability, the hybrid capacitor operated reliably over 400 cycles at the current density of 30 mA g^−1^ and showed minimal degradation in both electrochemical capacitance and CO_2_ adsorption capacity, with an average Coulombic efficiency of over 99% (Figure S12), which was comparable to reported symmetric supercapacitor systems.^15^ In contrast to measurements under pure CO_2_, electrochemical measurements of the hybrid capacitors under pure N_2_ and O_2_ showed no noticeable N_2_ or O_2_ pressure change during the charge−discharge processes (Figure S13), with average Coulombic efficiencies of over 97% and 93% over several cycles, respectively, demonstrating high CO_2_ selectivity, albeit with some electrochemical side reactions in the presence of O_2_. In the positive charging mode, the counter electrode which carries electrons was fully immersed by the electrolyte to avoid the direct reactions with O_2_ to some extent. These phenomena are also consistent with the proposed “ionic liquid-solid” mechanism and mirror previous discoveries on the CO_2_ selectivity of supercapacitors over O_2_ and N_2_.^4,15^ Here our entirely new asymmetric hybrid capacitor system showed enhanced CO_2_ adsorption capacities with good stability and high CO_2_ selectivity.

**Figure 3:**
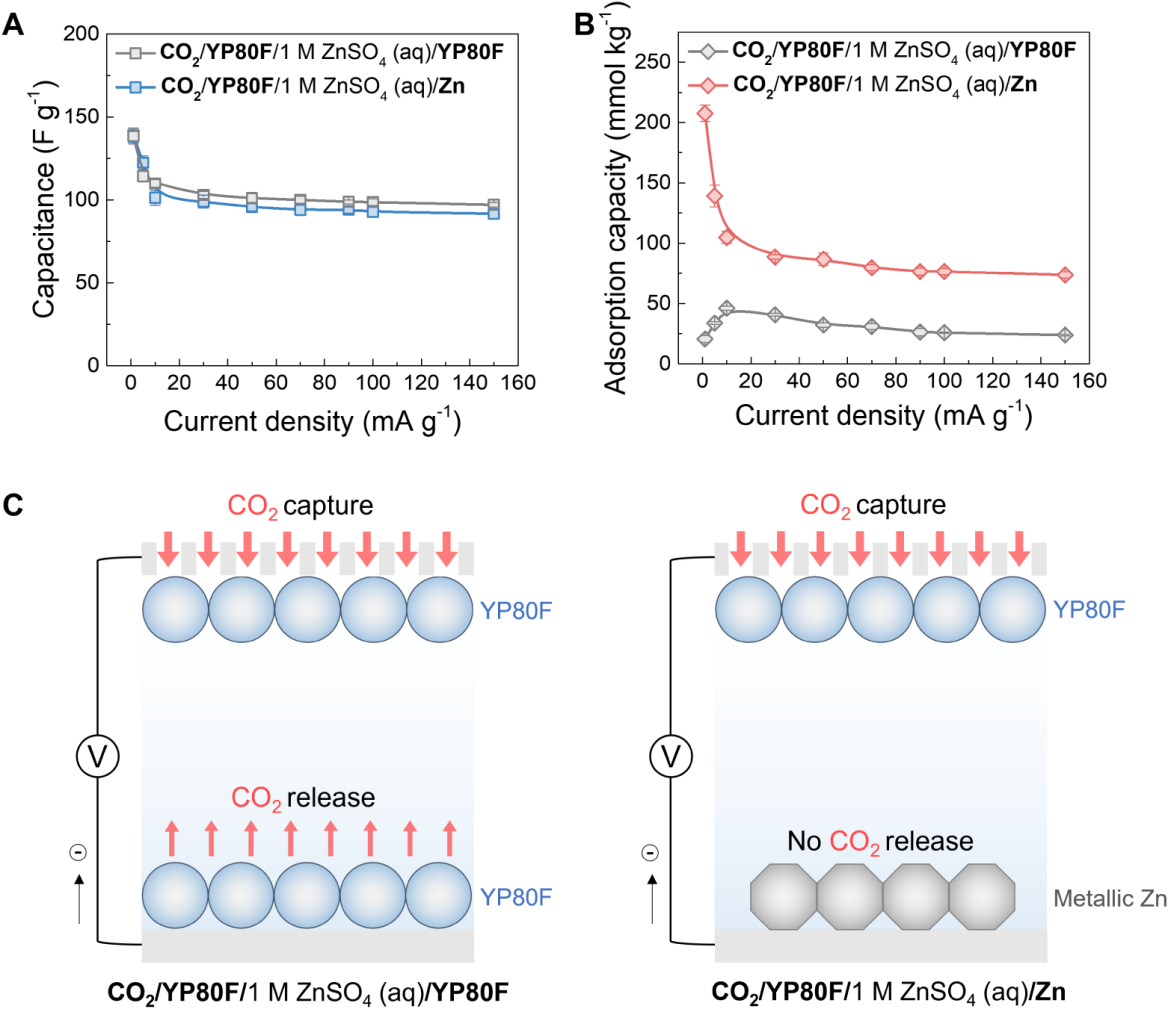
Kinetic performance of electrochemical CO_2_ capture of the hybrid capacitor and symmetric supercapacitor. Comparison of (A) the discharge capacitances of the activated carbon electrode and (B) CO_2_ adsorption capacities of the hybrid capacitor (noted as “CO_2_/YP80F/1 M ZnSO_4_ (aq)/Zn”) and symmetric supercapacitor (noted as “CO_2_/YP80F/1 M ZnSO_4_ (aq)/YP80F”) under CO_2_ at different current densities from 1 to 150 mA g^−1^ in the positive charging mode, with 5 min voltage/potential holds. (C) The schematic illustration of CO_2_ capture and release when the gas-exposed electrode obtains electrons in the symmetric supercapacitor (left) and the hybrid capacitor (right). All the discharge capacitances and CO_2_ adsorption capacities were normalized based on the active mass of the working electrode. The error was calculated using a 95% confidence interval with the Student’s *t* test.

The kinetic performance of charge storage and CO_2_ capture was then investigated by varying the current density from 1 to 150 mA g^−1^ (Figure 3A,B). The resulting electrochemical capacitances of the activated carbon in the hybrid capacitor were similar to those in the symmetric supercapacitor across a range of current densities from 1 to 150 mA g^−1^, decreasing steadily from 139 to 92 F g^−1^, which indicates similar capacitive charge storage kinetics in both systems (Figure 3A). However, significant differences in the CO_2_ uptake behavior were observed (Figure 3B). For the hybrid capacitor, the CO_2_ capacity rapidly decreased from 208 to 74 mmol_CO2_ kg^−1^ as the current density was increased from 1 to 150 mA g^−1^ (Figures 3B, S14 and S15). First, this suggests that CO_2_ capture is slower than charge storage, which is also supported by the experiments without the voltage/potential hold steps (Figures S8 and S9). In addition, this monotonic trend contrasts with that of the symmetric supercapacitor, which showed a decrease in the CO_2_ adsorption capacity at both high and low charging currents (Figure 3B). The decrease in the CO_2_ adsorption capacity at low current densities was also observed in our recent studies of symmetric supercapacitors,^15,24^ where we attributed this to competing CO_2_ capture and release processes at the two electrodes, while the CO_2_ adsorption capacity decrease at high current densities arises from a limited mass transport of CO_2_ into the cell.^24^ These findings support the idea that using metallic zinc as the electrolyte-immersed electrode effectively suppresses the competing processes (Figure 3C).

Similar trends were observed in the hybrid capacitors using varied electrodes and electrolytes (Figures S16−S24). We found that the activated carbon with a larger surface area, a combination of micro- and meso-pores and low oxygen functionalities generally performed best (Figure S18), aligning with our previous studies on electrode materials for SSA.^15^ Here YP80F activated carbon outperformed YP50F, which has similar surface chemistry but a smaller specific surface area, and O-YP80F (i.e., oxidized-YP80F), which has similar porosity but higher oxygen content (Figures S16−S18, Tables S2 and S3). We also found that the types of electrolyte anions and electrolyte concentrations might be critical factors (Figure S21). The traditional 1 M ZnSO_4_ (aq) electrolyte showed much better performance for electrochemical CO_2_ capture, compared to reported advanced electrolytes for Zn-ion batteries including 1 M Zn(CF_3_SO_3_)_2_ (aq) and 1 M Zn(CH_3_CO_2_)_2_ (aq) (Figure S21).^28,29^ Interestingly, the “water-in-salt” electrolyte composed of 1 M Zn(CH_3_CO_2_)_2_ (aq) and 20 M KCH_3_CO_2_ (aq) exhibited poorer electrochemical stability under CO_2_ than expected (Figure S22). This contrasts with reported works on “water-in-salt” electrolytes for Zn-ion batteries, which have demonstrated enlarged voltage windows.^30,31^ While more detailed mechanistic studies are needed to fully understand these phenomena, such investigations are beyond the scope of this work.

**Figure 4:**
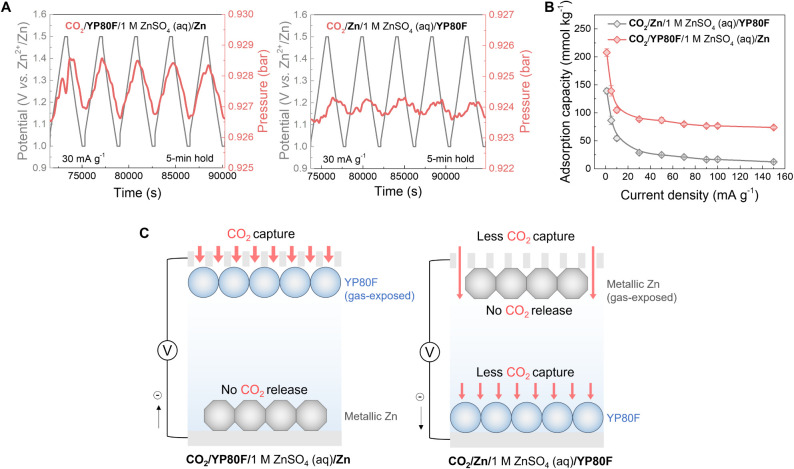
Impact of immersion of the activated carbon electrode on electrochemical CO_2_ capture performance in the hybrid capacitor. (A) Zoomed GCD curves (gray) and smoothed pressure curves (averaged every 100 s, red) of the hybrid capacitor with the normal configuration (noted as “CO_2_/YP80F/1 M ZnSO_4_ (aq)/Zn”) (left) and with the flipped configuration (noted as CO_2_/Zn/1 M ZnSO_4_ (aq)/YP80F) (right) under CO_2_ at the current density of 30 mA g^−1^ in the positive charging mode, with 5 min voltage/potential holds. (B) Comparison of CO_2_ adsorption capacities of the hybrid capacitor with the normal configuration and flipped configuration under CO_2_ at different current densities from 1 to 150 mA g^−1^ in the positive charging mode, with 5 min voltage/potential holds. (C) The schematic illustration of CO_2_ capture and release when the gas-exposed electrode obtains electrons in the hybrid capacitor with the normal configuration (left) and with the flipped configuration (right). In both schematics, CO_2_ capture occurs at the YP80F electrode, although this occurs at a lower rate in the flipped configuration. All the reported CO_2_ adsorption capacities were normalized based on the active mass of the working electrode. The error was calculated using a 95% confidence interval with the Student’s *t* test.

To investigate the impact of electrolyte immersion of the carbon electrode on electrochemical CO_2_ capture performance, we reversed the cell configuration of the hybrid capacitor, exposing the metallic zinc electrode to CO_2_ while immersing the activated carbon electrode in the electrolyte. The metallic zinc electrode was staggered when placed next to the meshed diffusion layer, minimizing the blocking effect of the metallic zinc electrode on gas diffusion. The flipped cell configuration was noted as “CO_2_/Zn/1 M ZnSO_4_ (aq)/YP80F”. Importantly, we still observed electrochemical CO_2_ adsorption and desorption in this flipped cell configuration, which followed the acquisition and loss of electrons at the carbon side, respectively (Figures 4A and S25), with the electrochemical capacitances of the activated carbon being similar in both configurations (Figure S26). The corresponding capacities of CO_2_ sorption continued to show a similar monotonic trend with current density as before (Figure 4B), suggesting that CO_2_ adsorption/desorption remains active at the activated carbon side, even when fully immersed in the electrolyte (Figure 4C, right). Thus, these measurements support the idea that competing CO_2_ adsorption and desorption processes exist at the two activated carbon electrodes in conventional symmetric supercapacitors.^24^ However, the CO_2_ capture/release of the hybrid capacitor became significantly reduced with the flipped cell configuration, particularly at higher current densities (e.g., 140 mmol_CO2_ kg^−1^ at 1 mA g^−1^ and 12 mmol_CO2_ kg^−1^ at 150 mA g^−1^) (Figure 4B), presumably because of the limited CO_2_ mass transport to the electrolyte-immersed carbon electrode compared to the gas-exposed electrode (Figure 4C, right). Hence, the CO_2_ adsorption previously observed in symmetric supercapacitors is indeed primarily a kinetic effect,^24^ driven by the differences in CO_2_ sorption rates at the two electrodes. In brief, we have further confirmed that electrochemical CO_2_ capture performance is enhanced in the hybrid capacitor because of the removal of the competing processes at the electrolyte-immersed electrode.

However, we cannot overlook the disadvantages of this hybrid capacitor compared to symmetric supercapacitors, which include the possible poor stability due to the dendrite formation at the zinc side after prolonged cycling,^32^ as well as the possible oxidation with trace amount of dissolved O_2_ in the aqueous electrolyte.^33^ Strategies developed for the stabilization of aqueous Zn-ion batteries can be used for reference to guide future studies on this new electrochemical CO_2_ capture device,^32^ and the use of relatively air-stable materials with similar functions to metallic zinc can be considered.

**Figure 5:**
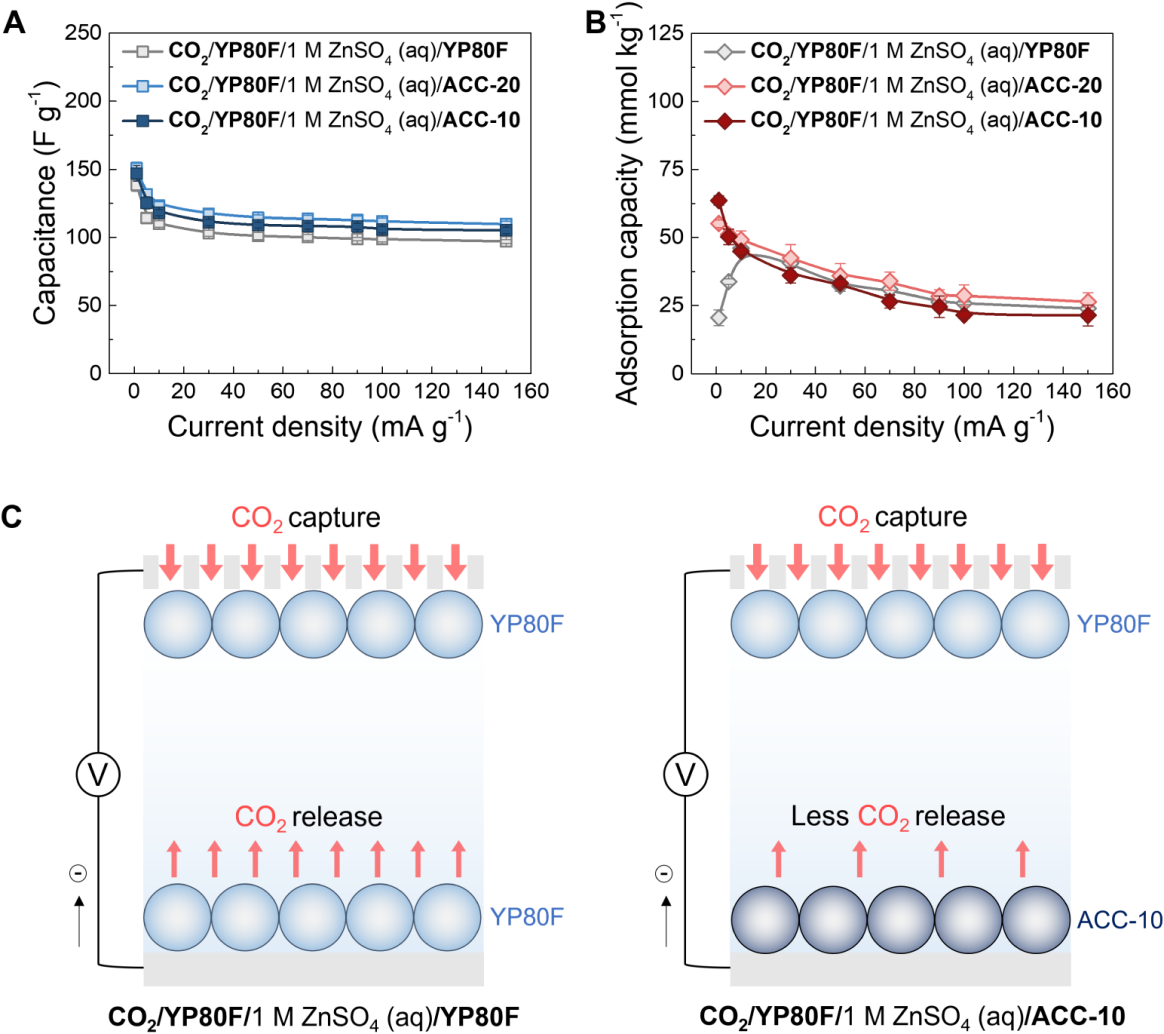
Generality of cell asymmetry in performance enhancement in supercapacitors. Comparison of (A) the discharge capacitances and (B) CO_2_ adsorption capacities of the symmetric supercapacitor (noted as “CO_2_/YP80F/1 M ZnSO_4_ (aq)/YP80F”) and asymmetric supercapacitors (noted as “CO_2_/YP80F/1 M ZnSO_4_ (aq)/ACC-10”, and “CO_2_/YP80F/1 M ZnSO_4_ (aq)/ACC-20”) under CO_2_ at different current densities from 1 to 150 mA g^−1^ in the positive charging mode, with 5 min voltage/potential holds. (C) The schematic illustration of CO_2_ capture and release when the gas-exposed electrode obtains electrons in the symmetric supercapacitor (left) and asymmetric supercapacitor (right). All the discharge capacitances and CO_2_ adsorption capacities were normalized based on the active mass of the working electrode. The error was calculated using a 95% confidence interval with the Student’s *t* test.

Inspired by the kinetic effects above, we hypothesized that using carbons with intrinsically slow CO_2_ sorption kinetics at the electrolyte-immersed side might further amplify the kinetic differences between the two sides in supercapacitors. In our previous study, we identified commercial activated carbons called ACC-10 and ACC-20, which have predominantly microporous structures with pore sizes smaller than 2 nm in diameter (Figure S27 and Table S2).^34^ These carbons have similar charge storage kinetics but slower CO_2_ sorption kinetics compared to YP80F, with ACC-10 showing the slowest CO_2_ sorption kinetics.^15^ Therefore, here we assembled the asymmetric supercapacitors designated as “CO_2_/YP80F/1 M ZnSO_4_ (aq)/ACC-10” and “CO_2_/YP80F/1 M ZnSO_4_ (aq)/ACC-20”, using ACC-10 and ACC-20 as the electrolyte-immersed counter electrode, respectively. Given the different electrochemical capacitances of ACC-10 and ACC-20 from YP80F, we adjusted the mass ratios of the electrodes (see Supporting Information), ensuring the equal distribution of potentials across the two different carbon electrodes in the asymmetric supercapacitors (Figure S6).

Regarding charge storage performance, the electrochemical capacitances of the asymmetric supercapacitors were similar to those of symmetric ones, ranging from 139 to 150 F g^−1^ at 1 mA g^−1^ and from 97 to 109 F g^−1^ at 150 mA g^−1^ (Figure 5A). However, the trend in CO_2_ adsorption capacities returned to monotonic patterns in the asymmetric supercapacitors (Figure 5B). Their CO_2_ adsorption capacities showed an obvious increase at slow charging rates (*e.g.,* 1 and 5 mA g^−1^). At 1 mA g^−1^, the CO_2_ adsorption capacity drastically increased by 200%, from 21 to 64 mmol_CO2_ kg^−1^ (Figure 5B), accompanied by noticeable changes in CO_2_ pressure at this current density (Figures S15, S28 and S29). These results are in support of our hypothesis that asymmetric carbon−carbon supercapacitors can enhance CO_2_ capture capacities (Figure 5C). However, at high charging rates (*e.g.,* 100 and 150 mA g^−1^), we did not observe such an increase in CO_2_ adsorption capacity, which contrasts to the behavior seen in the carbon−zinc hybrid capacitor (Figure 3B). This indicates the use of nonporous metallic zinc at the counter side can suppress the competing processes more effectively compared to the use of microporous carbons. When instead using 1 M Na_2_SO_4_ (aq) as the electrolyte (Figures S30 to S33), similar enhancements in CO_2_ adsorption capacities at slow charging rates (*e.g.,* 1, 5, and 10 mA g^−1^) were observed, with the highest reaching up to 114 mmol_CO2_ kg^−1^ at 10 mA g^−1^ using ACC-10. However, their electrochemical capacitances were still similar to each other and remained comparable even though a different electrolyte was used, with a range of 148 to 163 F g^−1^ at 1 mA g^−1^ and a range of 106 to 115 F g^−1^ at 150 mA g^−1^ (Figure S30). Here, the cations in the aqueous electrolytes appear to have a more pronounced effect on CO_2_ adsorption capacities than on electrochemical capacitances, possibly because of the different CO_2_ solubilities in these electrolytes.^35−37^ For the detailed effects of electrolyte compositions on symmetric supercapacitors, please refer to a previous work.^18^ Summarizing, with an asymmetric but all-carbon cell configuration, we further verify the generality of cell asymmetry in enhancing electrochemical CO_2_ capture performance, particularly at slow charging rates.

## Discussion

This work has established supercapacitor-battery hybrid systems as a new technology for electrochemical CO_2_ capture. Our activated carbon−zinc hybrid system captures CO_2_ when the capacitive electrode gains electrodes and releases CO_2_ when the capacitive electrode loses electrons, and shows significantly higher CO_2_ capture capacities compared with previous symmetric supercapacitor systems. Importantly, we uncover the previously overlooked competing processes between CO_2_ capture and release at the two activated carbon electrodes in supercapacitors, helping to explain previous observations that supercapacitive swing adsorption in symmetric supercapacitors is a kinetic effect. Hence, we propose a general methodology for enhancing electrochemical CO_2_ capture by breaking the symmetry present in traditional supercapacitors. For example, with the use of nonporous metallic zinc or microporous carbons at the electrolyte-immersed side, we increase overall electrochemical CO_2_ capture capacities by reducing CO_2_ release processes at the electrolyte-immersed side, when the gas-exposed carbon electrode captures CO_2_. It is worth noting that additional investigations are necessary to fully resolve the mechanistic complexities in this field. Different mechanisms including “ionic liquid-solid”, “molecular liquid-solid” or “pH-swing” may predominate under different experimental conditions, electrode structure, and electrolyte compositions. Overall, this work brings new insights into the mechanism of supercapacitive swing adsorption of CO_2_ and establishes a new approach for advancing electrochemical CO_2_ capture technologies.

## Data Availability

All data are available in the main text or the Supporting Information. All raw experimental data files are available in the Cambridge Research Repository, Apollo. DOI: 10.17863/CAM.114728.

## Supporting Information Available

Experimental details, electrochemical CO_2_ capture measurements, CV measurements, EIS measurements, N_2_ sorption isotherms, XPS spectra, tables of details on pore structures, and surface functional groups of all studied carbons (PDF)
