# Supplementary material for: Breaking Supercapacitor Symmetry Enhances Electrochemical Carbon Dioxide Capture
Source: J Am Chem Soc. 2025 Apr 29;147(19):16189–97. doi: 10.1021/jacs.5c00999 (PMC12082624; doi:10.1021/jacs.5c00999)
Supplement: Supplementary file 1 — ja5c00999_si_001.pdf [file ja5c00999_si_001.pdf]

Supporting Information

Breaking Supercapacitor Symmetry Enhances Electrochemical Carbon Dioxide Capture

Zhen Xu,[1,*] Xinyu Liu,[1] Grace Mapstone,[1] Zeke Coady,[1] Charles Seymour,[1] Selina E. Wiesner,[1,2] Svetlana Menkin,[1] Alexander C. Forse[1,*]

[1] Yusuf Hamied Department of Chemistry, University of Cambridge, Cambridge CB2 1EW, United Kingdom.

[2] Faculty of Chemistry and Pharmacy, Ludwig-Maximilians-Universität München, Munich 81377, Germany.

Corresponding author. Email: zx293@cam.ac.uk, acf50@cam.ac.uk

Materials and Methods

Electrode fabrication

Electrodes were prepared using activated carbons and polytetrafluoroethylene (PTFE) binder, maintaining a 95:5 weight ratio, respectively. The porous carbon powders employed were YP50F and YP80F (Kuraray). For the oxidation of YP80F, 400 mg of carbon powders were mixed with 15 mL of H_2O_2 solution (30 % (w/w) in H_2O, Sigma Aldrich) under magnetic stirring for 7 days. The resulting oxidized carbon powders (noted as O-YP80F) were washed with 250 mL deionized water 3 times and dried in an incubator under 60 °C overnight. Before the electrode fabrication, all the carbon materials were dried in a vacuum oven at 95°C overnight. For the electrode fabrication, around 200 mg carbon materials were dispersed in 5 mL of absolute ethanol (Sigma Aldrich) and combined with a PTFE dispersion (60 wt% dispersion in H_2O, Sigma Aldrich), followed by stirring for roughly an hour to attain a dough-like consistency after ethanol evaporation. The mixture was subsequently rolled on a glass sheet with a roller (0.25 mm thickness) to create a free-standing electrode. This electrode was transferred onto aluminum foil and dried in a vacuum oven at 95 °C overnight. Furthermore, commercially available free-standing microporous carbon cloth electrodes (ACC-10 and ACC-20, Kynol) were utilized. Before use, these materials were washed (soaked) with deionized water flow for 1 min and dried in a vacuum oven at 95 °C overnight. Circular electrodes with a diameter of 0.5 inches (around 12 mm) were cut out to achieve an approximate mass of 15 mg for CO_2 capture testing purposes. Metallic zinc disks with a diameter of around 12 mm were cut from the commercial zinc foil (0.62 mm thickness, Fisher Scientific Ltd).

Material characterization

The pore structures of carbon materials were tested using N_2 sorption isotherms (Anton Parr Autosorb iQ-XR) at 77 K. Before the testing, samples were degassed at 120 ºC under vacuum for 16 h. Brunauer–Emmett–Teller surface areas were calculated from isotherms using the BET equation, and pore size distributions were obtained using the quenched solid density functional theory (QSDFT) and slit pore model.[1] The surface chemistry of carbon materials was characterized using X-ray photoelectron spectroscopy (Thermo Fisher K-Alpha* XPS facility) with a monochromated Al-Kα X-ray source. Before the testing, samples were stuck onto the specific sample holders using conductive double-sided carbon tapes. Before the analysis of XPS, the samples were degassed under a high vacuum ($< 5 \times 10^{-7}$ bar) for 90 mins. Survey scans were measured using 200 eV pass energy, 1 eV step size and 200 ms (10 ms × 20 scans) dwell times and analyzed using the Avantage software. Atomic compositions were calculated and averaged according to the spectra acquired from 2-3 different spots on each sample. Nuclear magnetic resonance (NMR) spectroscopy experiments were carried out with a Bruker Avance Neo spectrometer in a Bruker 3.2 mm HXY triple resonance probe. Measurements were carried out at a magnetic field strength of 9.4 T, corresponding to a ^1H Larmor frequency of 400.1 MHz. All spectra were acquired with a 90º pulse-acquire sequence at an MAS speed of 5 kHz. The

90° pulse length was optimised for each sample. Recycle delays were set to > $5T_1$ for the CO_2 and HCO_3^- peaks for each sample to ensure measurements were quantitative, based on measurement of T_1 through inversion recovery experiments. ^{13}C NMR spectra were referenced relative to the ^{13}C CH resonance of adamantane at 37.78 ppm as a secondary reference to TMS.[2]

Three-electrode measurements

Three-electrode measurements were performed in the Swagelok cell as shown in Figure S7, where we used the working electrode (*i.e.,* YP80F, Diameter: 8 mm) to couple with different counter electrodes (*i.e.,* YP80F, ACC-10, ACC-20, and metallic zinc). Two GF/A separators (Whatman, Diameter: 10 mm) and 750 μL of 1 M $ZnSO_4$ (aq) electrolyte were used. Together with the Hg/HgO reference electrode (Alvatek), the cyclic voltammetry was conducted to monitor the corresponding potential changes of the working and counter electrodes at the scan rate of 2 mV s^{-1}.

Electrochemical CO_2 capture measurements

Electrochemical gas adsorption experiments were performed using a custom-designed gas cell at 303 K.[3] Electrochemical capacitors with a 1 M $ZnSO_4$ (aq) electrolyte were assembled within a coin cell with a meshed top case to allow gas access (SS316 CR2032, Cambridge Energy Solution). During coin cell assembly, the gas-exposed electrode (*i.e.,* YP80F, YP50F, O-YP80F, Diameter: 12 mm), the electrolyte-immersed electrode (*i.e.,* YP80F, YP50F, O-YP80F, ACC-10, ACC-20, and metallic zinc), two 0.5 mm stainless steel spacers, one conical spring, two GF/A separators (Whatman, Diameter: 20 mm) and 200 μL of 1 M $ZnSO_4$ (aq) electrolyte were used. After assembly, all components including electrodes in the meshed coin cell were firmly stacked together with a fixed total cell thickness of 3.2 mm. For symmetric supercapacitors, two identical carbon films with the same mass were used, and for asymmetric supercapacitors, the mass ratios of YP80F to ACC-10 and YP80F to ACC-20 were adjusted to 1.3 and 1.1, respectively, balancing the charges between two electrodes. For the flipped hybrid capacitors, the Zn electrode was staggered when placed next to the meshed diffusion layer, minimizing the blocking effects of the metallic zinc electrode on gas diffusion. For the symmetric zinc-zinc configuration, the cell was tested with the same under identical conditions, and the top zinc piece was also staggered when placed next to the meshed diffusion layer to allow CO_2 diffusion. After that, the meshed coin cell was inserted in the gas cell with the mesh side facing the gas reservoir, followed by the filling of the gas reservoir with pure CO_2 (99.80% purity, BOC), N_2 (99.998% purity, BOC) or O_2 (99.5% purity, BOC). For air-to-CO_2 exchange in the gas reservoir, a gas manifold was employed (Figure S1). To prevent electrolyte evaporation, the cell was subjected to a static vacuum. Subsequently, the valve closest to the cell was shut, and the gas manifold was dosed with CO_2 at around 1.3 bar. The decreased pressure in the gas cell aids the mixture of the gas reservoir with CO_2 from the manifold upon opening the cell valve. Then the cell valve was closed, and the manifold returned to dynamic vacuum. This dosing process was

iterated 4 more times to establish an approximately pure CO_2 headspace. The same dosing protocols were employed for air-to-N_2 and air-to-O_2 exchanges.

A potentiostat (VSP-3e and VMP-3e, Biologic) was used to conduct the electrochemical testing of gas cells including the galvanostatic charge and discharge measurement (GCD), cyclic voltammetry (CV) and electrochemical impedance spectroscopy (EIS). The gas adsorption or desorption was measured in a 30 °C incubator (SciQuip Incu-80S) by monitoring the gas reservoir pressure of the electrochemical gas cell with a pressure transducer (PX309-030A5V, Omega). The noise of the pressure transducer is at the level of 0.1 mbar, and the signal-to-noise ratio is over 5, which indicates a reasonable sensitivity of the pressure sensor. We also averaged the pressure data every 100 seconds to further decrease the effect of random pressure noise. In addition, we validated the pressure transducer using the two additional pressure sensors (MKS PDR2000 Dual Capacitance Manometer) on the gas manifold with accuracy at the level of 0.01 mbar (Figure S1), ensuring high measurement accuracy and reliability.

Considering the challenges associated with equilibration time in static gas methods (*i.e.,* electrochemical gas capture measurements without gas flow) and the slower gas diffusion rates, all gas cells were pre-cycled under 1 mV s^{-1} for 20 cycles (~ 8 hours) during which time CO_2 continued to equilibrate with the cell. The 1-hour rest before the regular GCD measurement was associated with a horizontal pressure baseline, which indicates the established equilibrium of the whole system after pre-cycling (Figure S10). All the electrochemical CO_2 capture measurements were repeated using at least two independent cells to confirm the reproducibility (Figure S11).

Calculations

The specific discharge capacitance values of the working electrodes were calculated from GCD (galvanostatic charge-discharge) measurements according to Equation S1, as follows:[4]

(1) $C_{electrode} = n\frac{I\Delta t}{m\Delta U}$

where $C_{electrode}$ (F g^{-1}) refers to the specific discharge capacitance of the working electrodes, n equals to 2 for supercapacitors and 1 for hybrid capacitors, I (A) is the constant charge/discharge current, ΔU (V) is the change range of the cell voltage (for the hybrid capacitor, it is the change range of the potential), m (g) is the mass of active material (*i.e.,* activated carbon) loaded on the working electrodes, and Δt (s) is the discharge time under galvanostatic discharging. The cell voltage of the whole device is:

(2) $\Delta U = U_{max} - IR_{drop} - U_{min}$

where U_{max} and U_{min} are the maximum and minimum voltage applied, respectively.

The input/output energy values of the full device were obtained using Equation S3, as follows [3]:

(3) $E_{in/out} = \frac{I}{m}\int_{t_1}^{t_2} U(t)dt + \frac{U}{m}\int_{t_3}^{t_4} I(t)dt$

where $E_{in/out}$ (kJ kg^{-1}) is the input/output energy normalized by the active mass of the working electrode. I (A) is the constant charge/discharge current, m (g) is the active mass of the working electrode, and U (V) is the constant voltage/potential. U(t) (V) is the voltage/potential that changes with time under galvanostatic discharging or charging, t_1 (s) is the start time of galvanostatic charge/discharge processes, and t_2 (s) is the end time. I(t) (A) is the current that changes with time during voltage/potential hold, t_3 (s) is the start time of voltage/potential, and t_4 (s) is the end time.

The Coulombic efficiency (CE, %) was calculated to evaluate the reversibility of the stored charges using equation S4, as follows:

(4) $CE = \frac{\int_{t_3}^{t_4} I(t)dt}{\int_{t_1}^{t_2} I(t)dt} \times 100\%$

where I(t) (A) is the current that changes with time. t_1 and t_2 (s) are the start time and end time of charge processes, respectively. In addition, t_3 and t_4 (s) are the start time and end time of discharge processes, respectively.

The specific gravimetric CO_2 adsorption capacity (C_{CO2}, $mmol_{CO2}$ kg^{-1}) was calculated by taking the difference between the maximum and minimum peaks of the gas amount (Δn, mol) in the reservoir and normalized by the active mass of the working electrode. An average of two minimum points is used to avoid the error from the irreversible pressure changes when calculating adsorption capacity. This was converted from the pressure transducer data (smoothed every 100 seconds for all experiments) using the ideal gas law:[3]

(5) $n = \frac{pV}{RT}$

(6) $\Delta n = \frac{n_{max_1} + n_{max_2}}{2} - n_{min}$

(7) $C_{CO_2} = \frac{\Delta n}{m} \times 10^6$

where p, V and T are the pressure (Pa, 10^{-5} bar), volume (m^3, 10^6 mL) and temperature (K) respectively, and n is the gas amount (mol). R is the ideal gas constant (8.31451 m^3 Pa mol^{-1} K^{-1}), and m (g) is the active mass of the working electrode. In a completed cycle of CO_2 adsorption and desorption, n_{max1} and n_{max2} represent the two maximum peaks of the gas amount, and n_{min} represents the minimum peak of the gas amount. The overall adsorption capacity was taken as the mean of adsorption capacities for 6 cycles, and the error was calculated using a 95% confidence interval with the Student's t-test of performance from cycle to cycle at the same current density.

The volume of the gas reservoir (V_1, mL) in the gas cell was calculated during the process of dosing CO_2 into the cell, based on pressure measurements of the added gas using Boyle's Law ($P_1V_1 = P_2V_2$). For each gas cell, the volume in a section of pipe between two valves was

known from a prior calibration (*A* mL) (Figure S1). First, the gas between two valves was removed using a vacuum, and the gas amount in the main gas reservoir was proportional to P_1V_1, where P_1 (bar) was read from the pressure sensor (Figure S10). After that, we allowed gas to enter the evacuated portion of the known volume (*A* mL). During this process, the total amount of gas remained the same (therefore, $P_1V_1 = P_2V_2$) but the pressure decreased to P_2 (bar) (Figure S10). As V_2 (mL) was equal to the sum of V_1 (mL) and *A* (mL), the volume of the reservoir (V_1, mL) was calculated using Equation S8:

(8) $V_1 = \frac{P_2A}{P_1-P_2}$

This step was also used as the leaking test to make sure there was no leaking of the gas cell. If the gas cell leaks, the pressure will not be maintained at the low pressure of P_2 (bar) < 1 bar (Figure S10).

The specific electrical energy consumption (E, kJ mol_{CO2}^{-1}) were calculated using equation S9, as follows:[5,6]

(9) $E = \frac{E_{in}-E_{out}}{C_{CO_2}} \times 10^3$

Figures and Tables

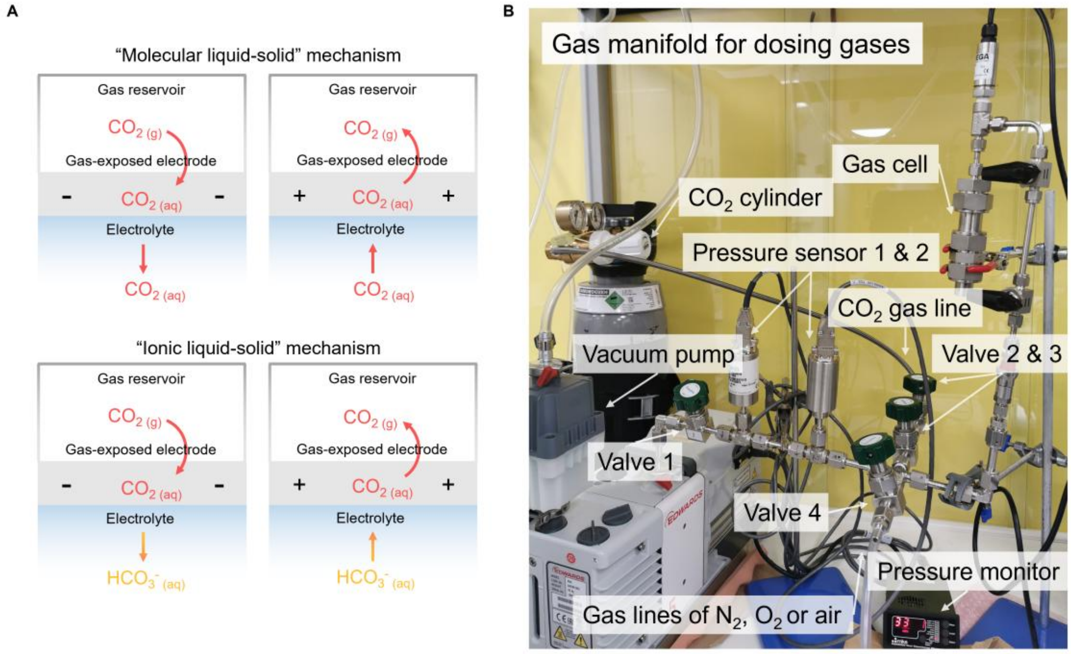

Figure S1. The mechanistic models and the setup of the gas manifold for dosing gases. (**A**) The schematic illustration of "molecular liquid-solid" mechanism and "ionic liquid-solid" mechanism. The curved arrow reflects the observed pressure change from experiments, while the straight arrows show the hypothesized CO_2-derived species in the electrolyte. (**B**) A photo of the gas manifold for dosing gases into the electrochemical gas cell, where Valve 1 was used to control the connection between vacuum pump and gas cell, Valves 2 & 3 were used to control the connection between CO_2 cylinder and gas cell, and Valve 4 was used to control the connection between other gas cylinders and gas cell.

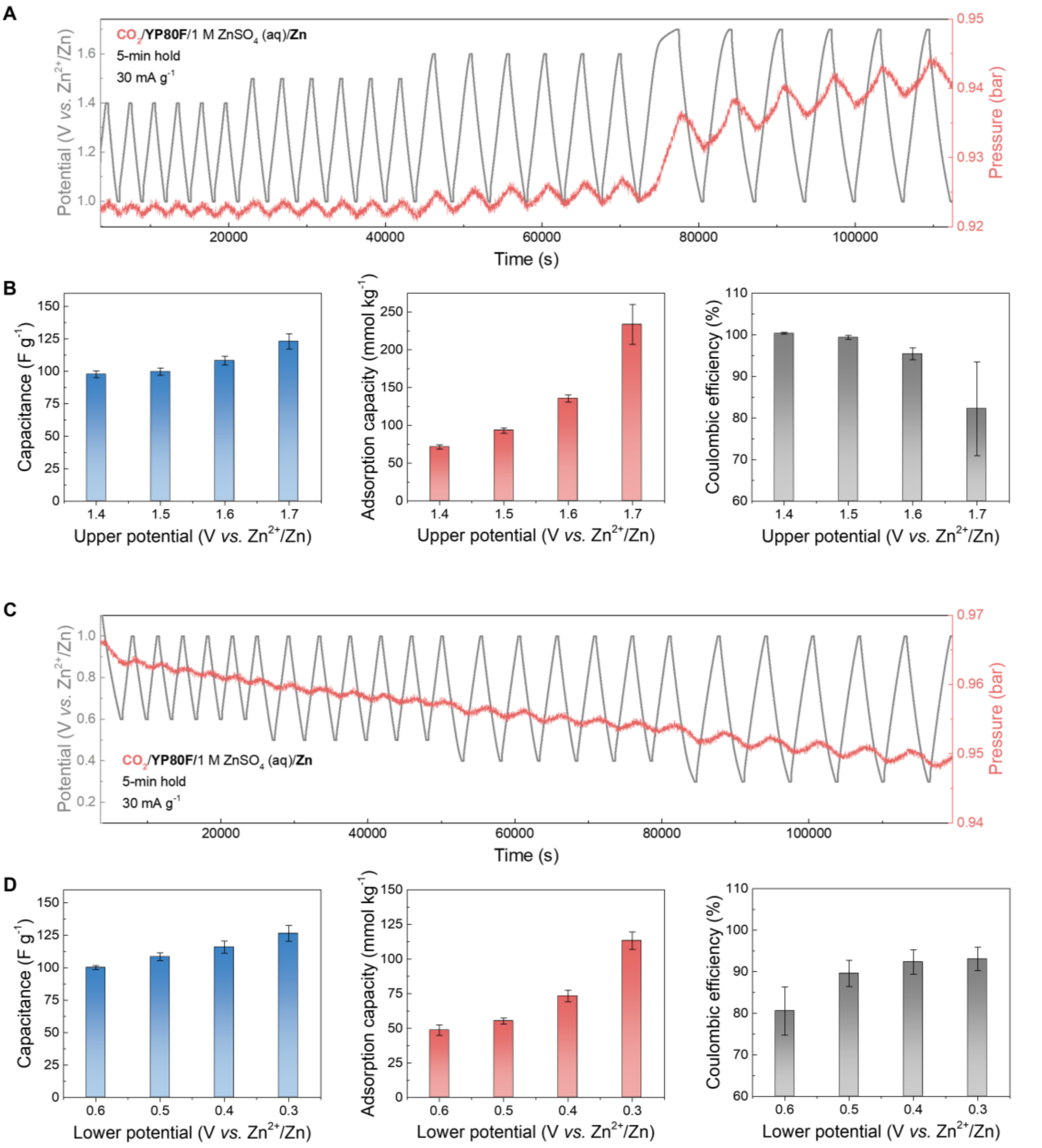

Figure S2. Exploring the potential limits of the hybrid capacitor under CO_2. (**A**) Overall GCD curves (grey) and corresponding pressure curves (red) of the device with the asymmetric cell configuration of "CO_2/YP80F/1 M $ZnSO_4$ (aq)/Zn" at the current density of 30 mA g^{-1} in different positive charging modes, all with 5-min voltage/potential holds. (**B**) Comparison of the corresponding discharge capacitances, CO_2 adsorption capacities and Coulombic efficiencies of the working electrode with different upper potentials. (**C**) Overall GCD curves (grey) and corresponding pressure curves (red) of the device with the asymmetric cell configuration of "CO_2/YP80F/1 M $ZnSO_4$ (aq)/Zn" at the current density of 30 mA g^{-1} in different negative charging modes, all with 5-min voltage/potential holds. (**D**) Comparison of the corresponding discharge capacitances, CO_2 adsorption capacities and Coulombic efficiencies of the working electrode with different lower potentials. All the discharge capacitances and CO_2 adsorption capacities were normalized based on the active mass of the working electrode. All the discharge capacitances and CO_2 adsorption capacities were

normalized based on the active mass of the working electrode. The error was calculated using a 95% confidence interval with the Student's t-test. Notes: Significant irreversible pressure increases, and decreases in Coulombic efficiencies, were seen for potentials of 1.6 V vs. Zn^{2+}/Zn. Upper potentials were therefore limited to 1.5 V in this study. Significant irreversible pressure increases, and decreases in Coulombic efficiencies, were seen in negative charging modes. The positive charging modes were therefore used in this study.

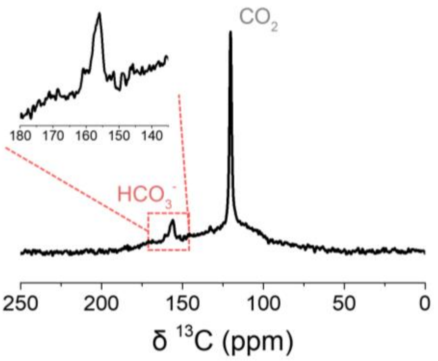

Figure S3. Solid-state nuclear magnetic resonance (NMR) measurement of $^{13}CO_2$-dosed carbon electrode demonstrates bicarbonate formation. ^{13}C solid-state NMR spectroscopy (9.4 T, 5 kHz MAS) of YP80F soaked in 1 M $ZnSO_4$ (aq) dosed with $^{13}CO_2$ (gas). Notes: The left-hand resonance at around 156 ppm corresponds to $^{13}CO_2$-derived $H^{13}CO_3^-$ ions.[6] The broad, asymmetrical peak shape indicates that $H^{13}CO_3^-$ ions exist inside and outside the carbon pores.[7,8] The right-hand peak of around 120 ppm is assigned to $^{13}CO_2$ inside the carbon pores,[9] and the broad feature underneath arises from activated carbon YP80F.

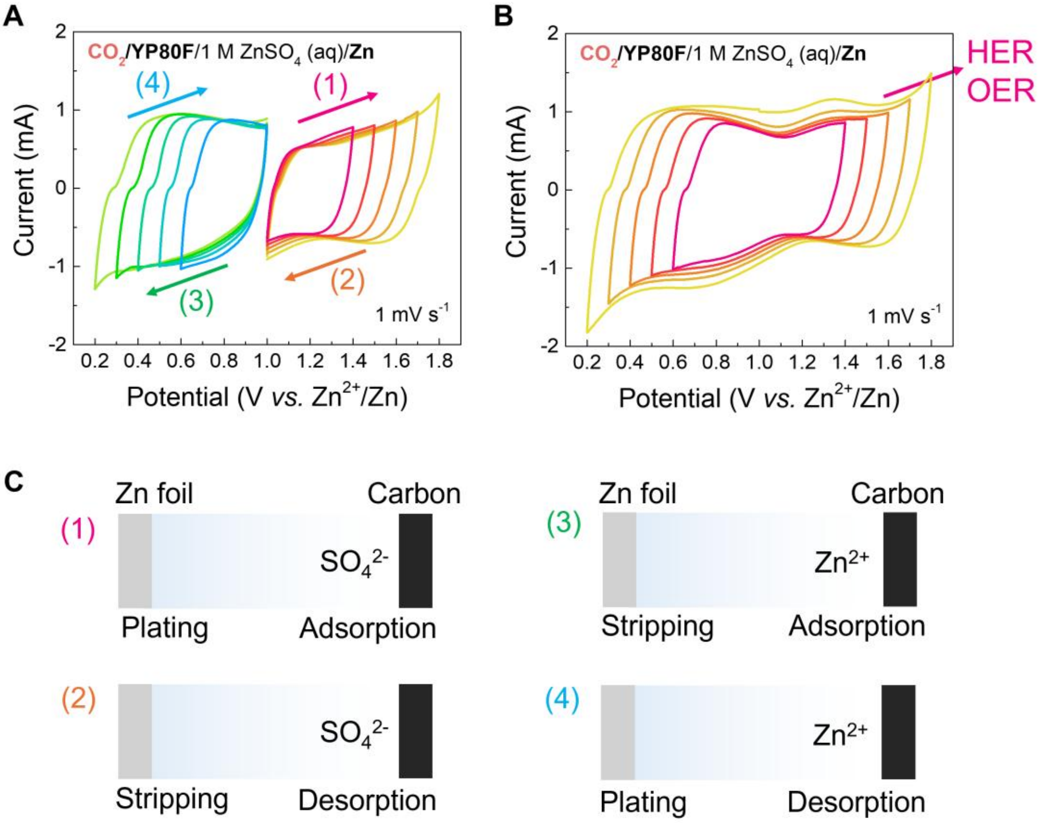

Figure S4. CV measurements of the hybrid capacitor under CO_2. (**A**) CV curves of the device with the asymmetric cell configuration of CO_2/YP80F/1 M $ZnSO_4$ (aq)/Zn at the scan rate of 1 mV s^{-1} in the positive and negative charging modes. (**B**) CV curves of the device with the asymmetric cell configuration of "CO_2/YP80F/1 M $ZnSO_4$ (aq)/Zn" at the scan rate of 1 mV s^{-1} in the switching charging mode which switches between the positive and negative charging modes with a full potential range. (**C**) Schematic illustration of the corresponding electrochemical behaviors at the working and counter electrodes in the positive and negative charging modes.

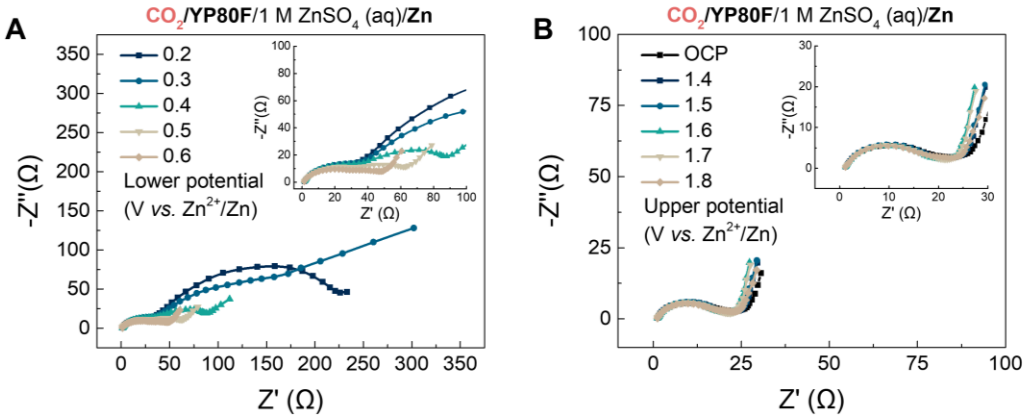

Figure S5. EIS measurements of the hybrid capacitor under CO_2. EIS curves of the device with the asymmetric cell configuration of "CO_2/YP80F/1 M $ZnSO_4$ (aq)/Zn" using the frequency ranging from 0.01 to 100k Hz at (**A**) negatively charged states and (**B**) positively charged states.

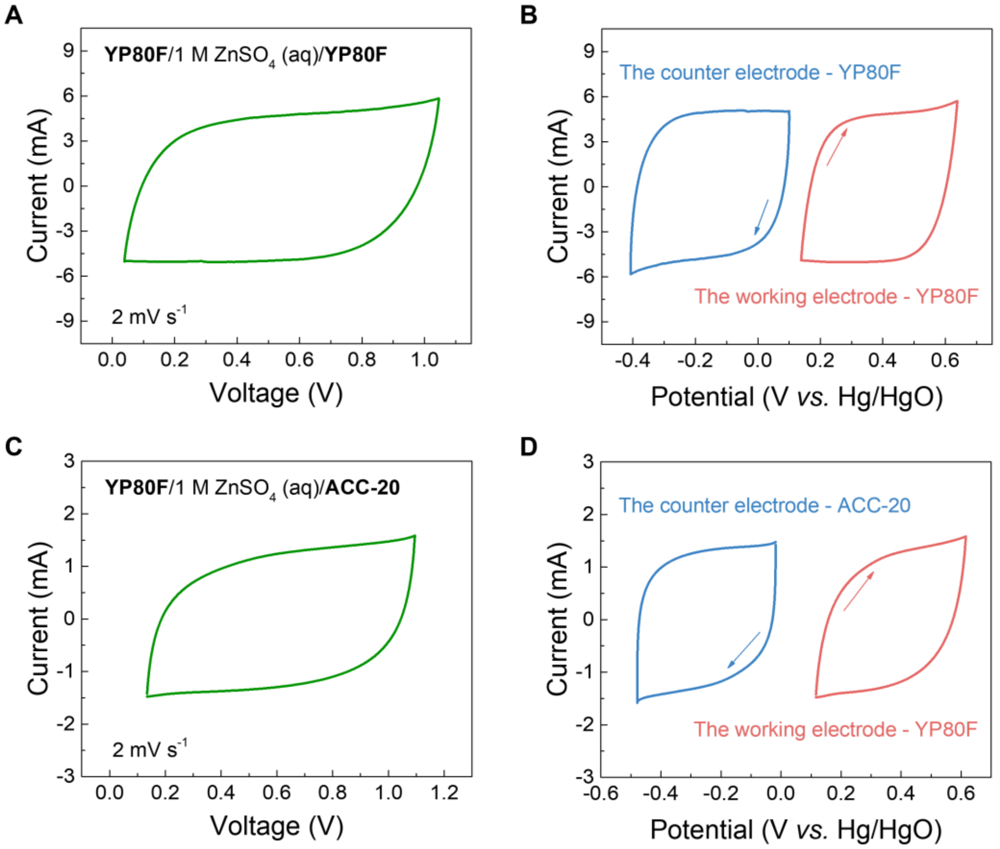

Figure S6. Three-electrode cyclic voltammetry measurements of supercapacitors. (**A**) Cyclic voltammetry (CV) of the device with the symmetric cell configuration of "YP80F/1 M $ZnSO_4$ (aq)/YP80F" in the positive charging mode at the scan rate of 1 mV s^{-1}. (**B**) The potential (*vs.* Hg/HgO) changes of the working electrode (red) and the counter electrode (blue) during the CV measurement when the YP80F working electrode is positively charged to 0.5 V *versus* open-circuit potential. (**C**) Cyclic voltammetry (CV) of the device with the asymmetric cell configuration of "YP80F/1 M $ZnSO_4$ (aq)/ACC-20" in the negative charging mode at the scan rate of 1 mV s^{-1}. (**D**) The potential changes of the working electrode (red) and the counter electrode (blue) during the CV measurement when the YP80F working electrode was positively charged to 0.5 V *versus* open-circuit potential.

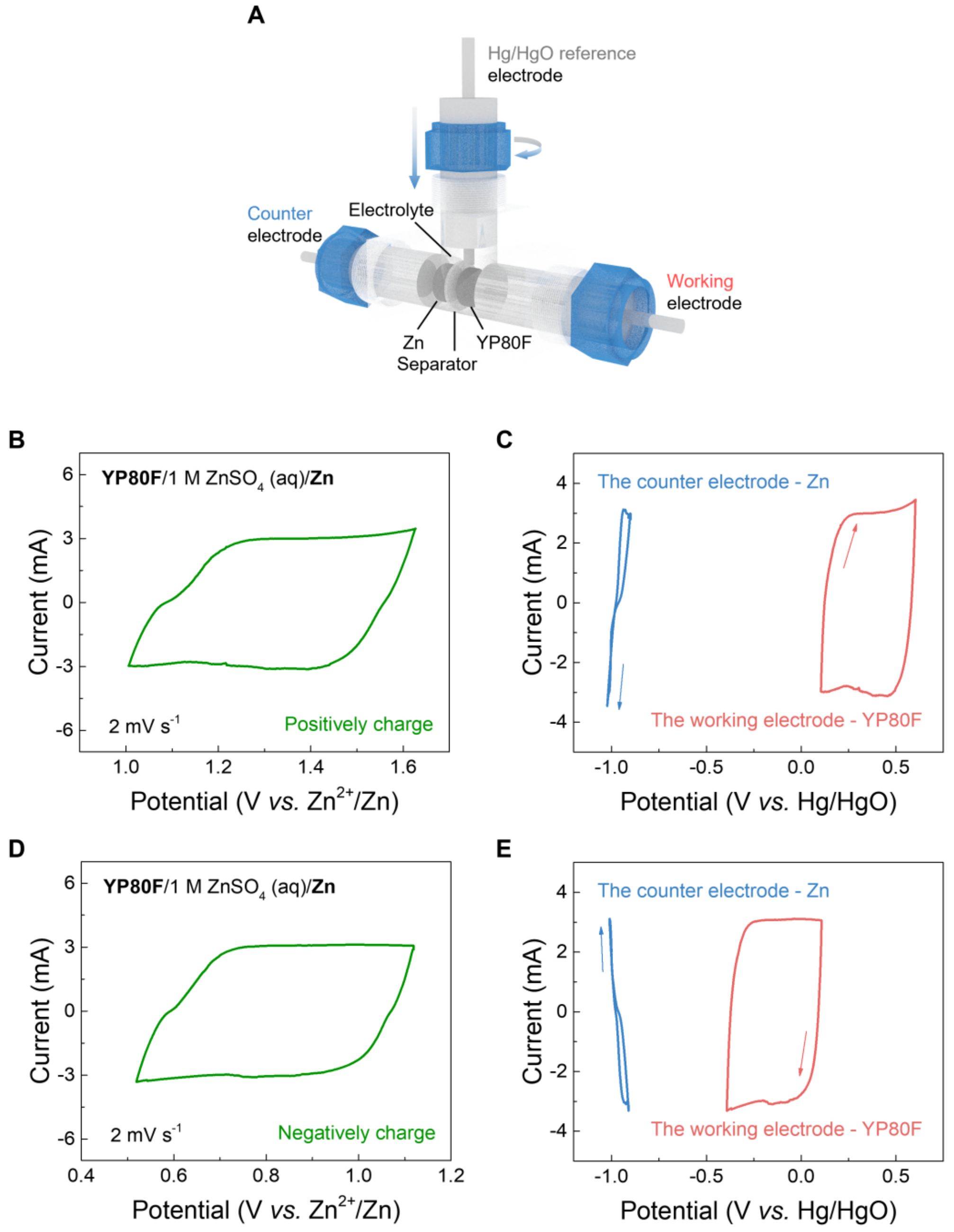

Figure S7. Three-electrode measurements of hybrid capacitors. (**A**) Schematic illustration of the three-electrode Swagelok cell setup using the YP80F working electrode, the Zn counter electrode and the Hg/HgO reference electrode. (**B**) Cyclic voltammetry (CV) of the device with the asymmetric cell configuration of "YP80F/1 M $ZnSO_4$ (aq)/Zn" in the positive charging mode at the scan rate of 1 mV s^{-1}. (**C**) The potential changes of the working electrode (red) and the counter electrode (blue) during the CV measurement when the YP80F working electrode is positively charged to 0.5 V *versus* open-circuit potential. (**D**) Cyclic voltammetry (CV) of the device with the asymmetric cell configuration of "YP80F/1 M $ZnSO_4$ (aq)/Zn" in the negative charging mode at the scan rate of 1 mV s^{-1}. (**E**) The potential changes of the working electrode (red) and the counter electrode (blue) during the CV measurement when the YP80F working electrode was negatively charged to –0.5 V *versus* open-circuit potential. Notes: The shapes of

the CV curves support the redox plating/stripping behaviors at the zinc side, and the electrochemical double-layer capacitive behaviors at the carbon side. In the three-electrode measurements with a limited voltage window (Figure S7), no noticeable redox peaks corresponding to HER or OER can be observed. Additionally, the carbon electrode can provide some buffering capacity against local pH changes during operation according to our recent work.[10] Future studies employing specialized techniques such as in-situ surface-enhanced Raman spectroscopy or pH-sensitive fluorescent probes could potentially provide insights into local pH variations during operation, but such analyses were beyond the scope of the present work.

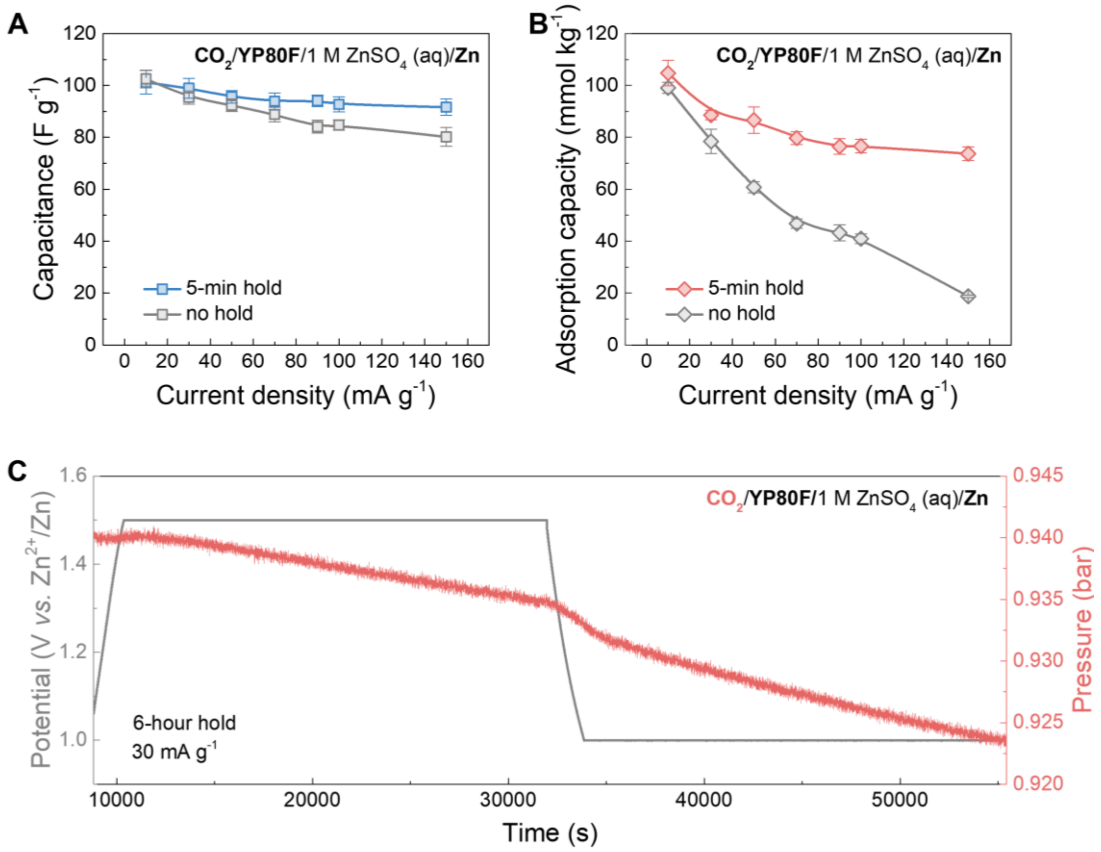

Figure S8. The effects of voltage/potential hold step on the performance of hybrid capacitors under CO_2. Comparison of (**A**) the discharge capacitances of the activated carbon electrode and (**B**) CO_2 adsorption capacities of the device with the asymmetric cell configuration of "CO_2/YP80F/1 M $ZnSO_4$ (aq)/Zn" at different current densities from 10 to 150 mA g^{-1} in the positive charging mode, with and without 5-min voltage/potential holds. (**C**) Overall GCD curves (grey) and corresponding pressure curves (red) of the device with the asymmetric cell configuration of "CO_2/YP80F/1 M $ZnSO_4$ (aq)/Zn" at the current density of 30 mA g^{-1}, with a voltage hold of 6 h. All the discharge capacitances and CO_2 adsorption capacities were normalized based on the active mass of the working electrode. The error was calculated using a 95% confidence interval with the Student's t-test.

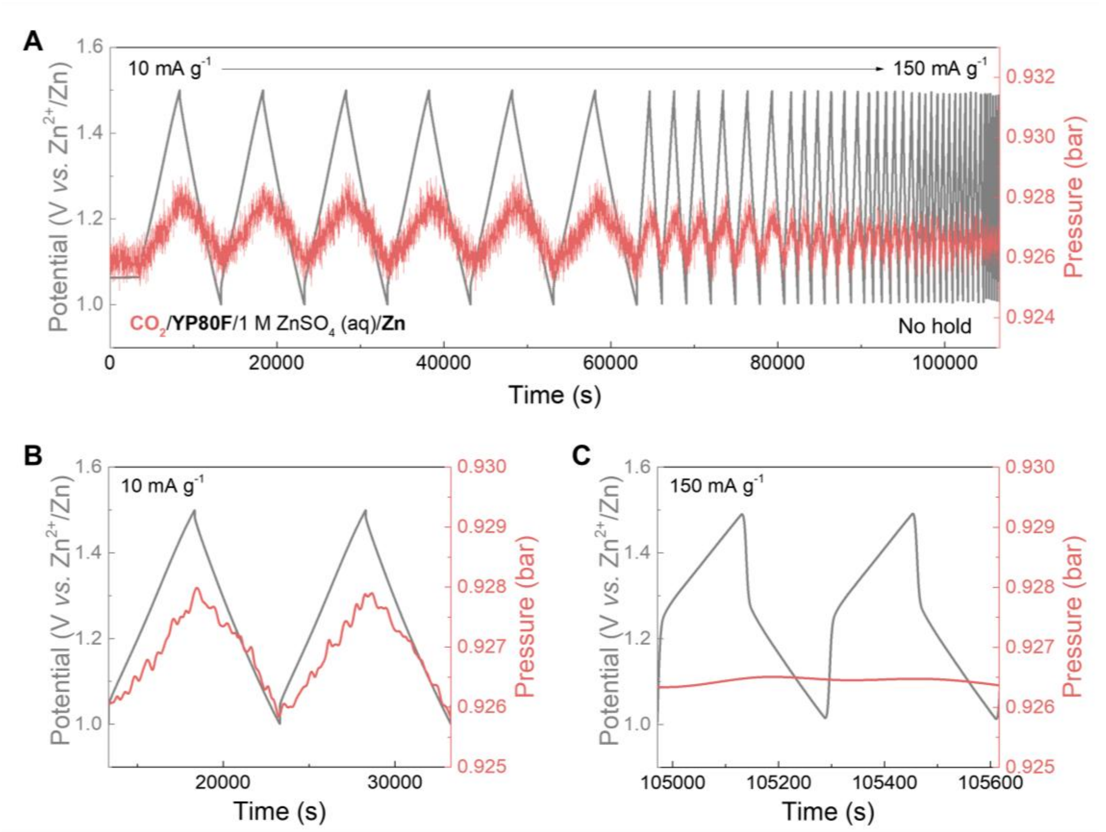

Figure S9. Electrochemical CO_2 capture measurements of the hybrid capacitor under CO_2 without voltage/potential hold. (**A**) Overall GCD curves (grey) and corresponding pressure curves (red) of the device with the asymmetric cell configuration of "CO_2/YP80F/1 M $ZnSO_4$ (aq)/Zn" at different current densities from 10 to 150 mA g^{-1} in the positive charging mode, all without voltage/potential hold. Zoomed GCD curves (grey) and smoothed pressure curves (averaged every 100 sec, red) of the device with the asymmetric cell configuration of "CO_2/YP80F/1 M $ZnSO_4$ (aq)/Zn" at the current densities of (**B**) 10 mA g^{-1} and (**C**) 150 mA g^{-1} in the positive charging mode, without voltage/potential hold. Notes: Without voltage/potential hold, we observed obvious IRdrop in GCD curves at the fast charging conditions.

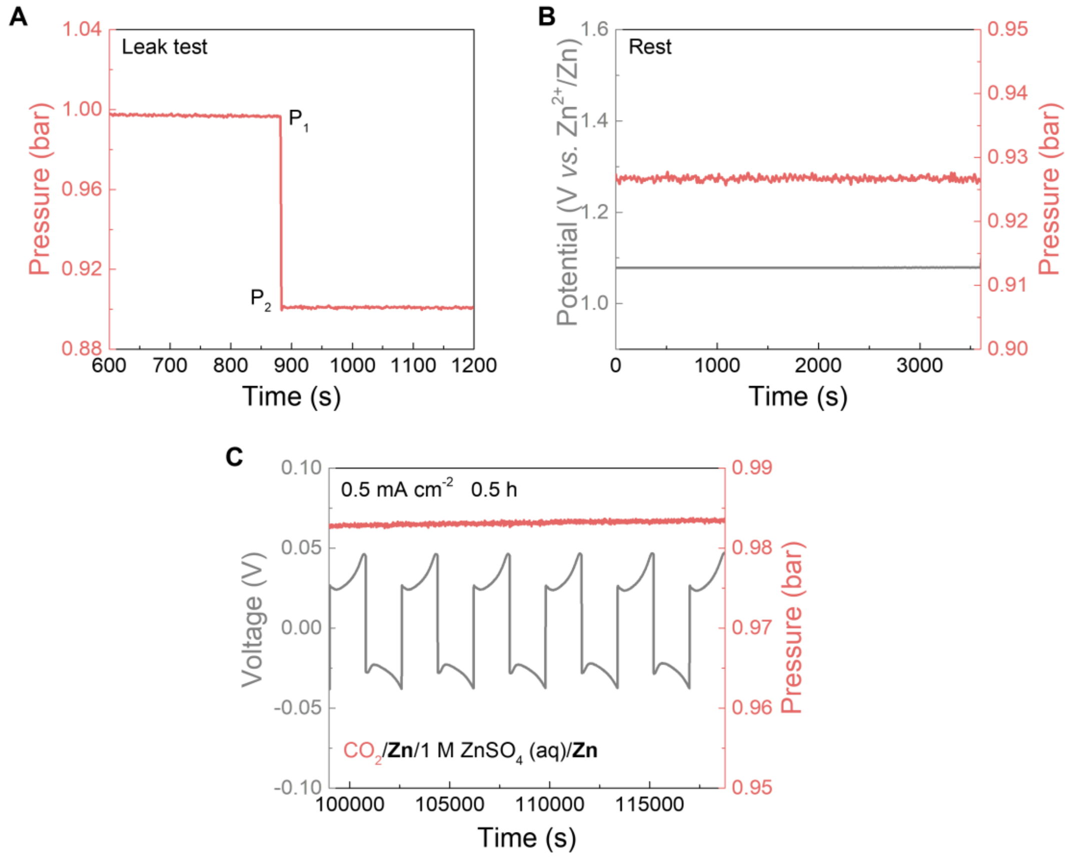

Figure S10. Leak tests and rest before GCD measurements & Zn-Zn symmetric cell measurements. (**A**) Pressure curves (red) of the device with the asymmetric cell configuration of "CO_2/YP80F/1 M $ZnSO_4$ (aq)/Zn" before and after gas was allowed to enter the evacuated portion between the two valves of the electrochemical gas cell. (**B**) Zoomed potential curves (grey) and pressure curves (red) of the device with the asymmetric cell configuration of "CO_2/YP80F/1 M $ZnSO_4$ (aq)/Zn" during the rest before testing. (**C**) Overall GCD curves (grey) and corresponding pressure curves (red) of the device with the symmetric cell configuration of "CO_2/Zn/1 M $ZnSO_4$ (aq)/Zn" at the current density of 0.5 mA cm^{-2} and the capacity of 0.25 mAh cm^{-2} (the top zinc piece was shifted to allow CO_2 diffusion).

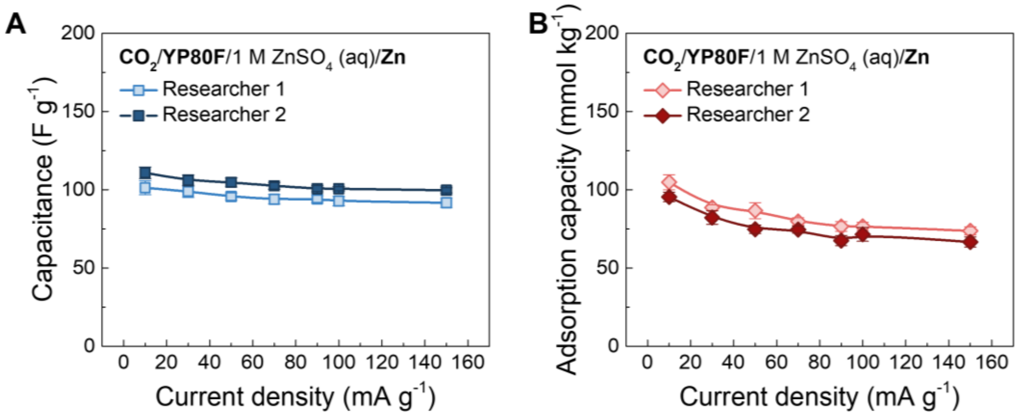

Figure S11. Evidence of reproducibility based on the measurements conducted by independent researchers. Comparison of (**A**) the discharge capacitances and (**B**) CO_2 adsorption capacities of the device with the asymmetric cell configuration of "CO_2/YP80F/1 M $ZnSO_4$ (aq)/Zn" at different current densities from 10 to 150 mA g^{-1} in the positive charging mode with 5-min voltage/potential holds, conducted by independent researchers. All the discharge capacitances and CO_2 adsorption capacities were normalized based on the active mass of the working electrode. The error was calculated using a 95% confidence interval with the Student's t-test.

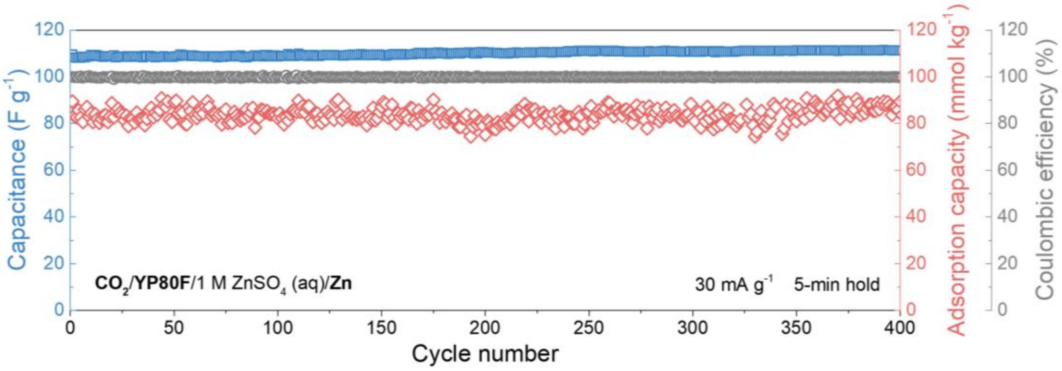

Figure S12. Prolonged cycling measurement of the hybrid capacitor under CO_2. Long cycling performance of the device with the asymmetric cell configuration of "CO_2/YP80F/1 M $ZnSO_4$ (aq)/Zn" including the discharge capacitances, CO_2 adsorption capacities and Coulombic efficiencies at the current density of 40 mA g^{-1} in the positive charging mode, with 5-min voltage/potential holds. All the discharge capacitances and CO_2 adsorption capacities were normalized based on the active mass of the working electrode. Note: The high average Coulombic efficiency (>99.8 % at 30 mA g^{-1}, Figure S12) of our hybrid capacitor system indicates minimal side reactions during normal operation. If significant water decomposition were occurring, we would expect much lower Coulombic efficiencies due to the irreversible nature of these reactions. In summary, by adjusting voltage window and cycling rate, we have furthest eliminated the interference from water decomposition.

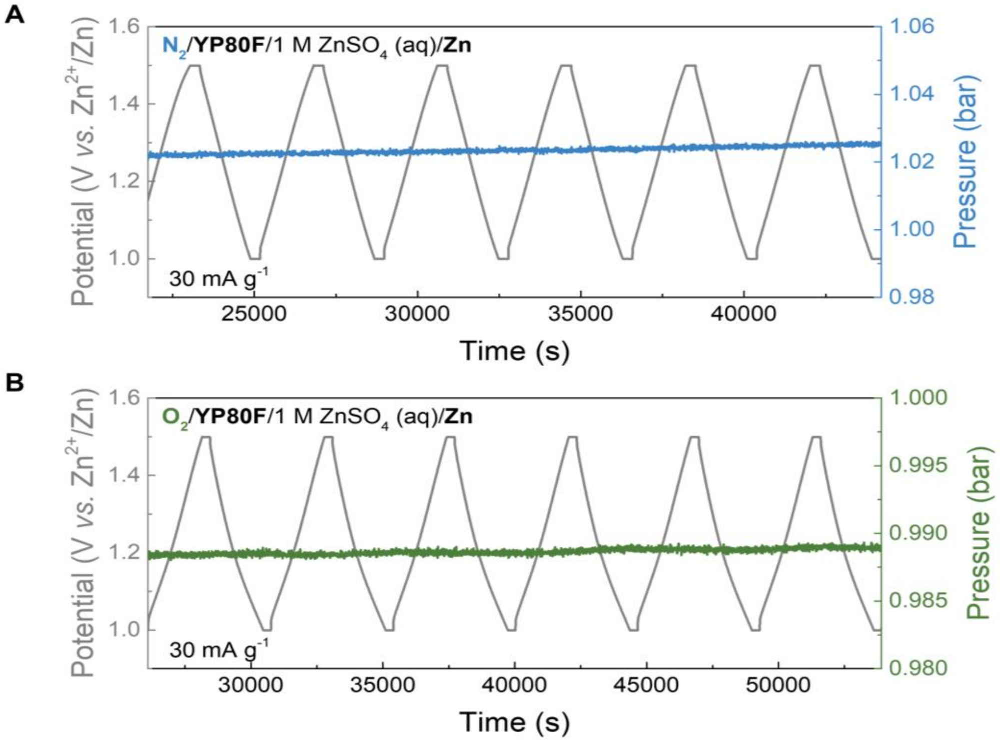

Figure S13. Initial evidence of CO_2 selectivity over N_2 and O_2. (**A**) Overall GCD curves (grey) and corresponding N_2 pressure curves (blue) of the device with the asymmetric cell configuration of "CO_2/YP80F/1 M $ZnSO_4$ (aq)/Zn" at the current density of 30 mA g^{-1} in the positive charging mode, with 5-min voltage/potential holds. (**B**) Overall GCD curves (grey) and corresponding N_2 pressure curves (green) of the device with the asymmetric cell configuration of "CO_2/YP80F/1 M $ZnSO_4$ (aq)/Zn" at the current density of 30 mA g^{-1} in the positive charging mode, with 5-min voltage/potential holds. Notes: No obvious pressure changes were observed except for the noise from the pressure sensor.

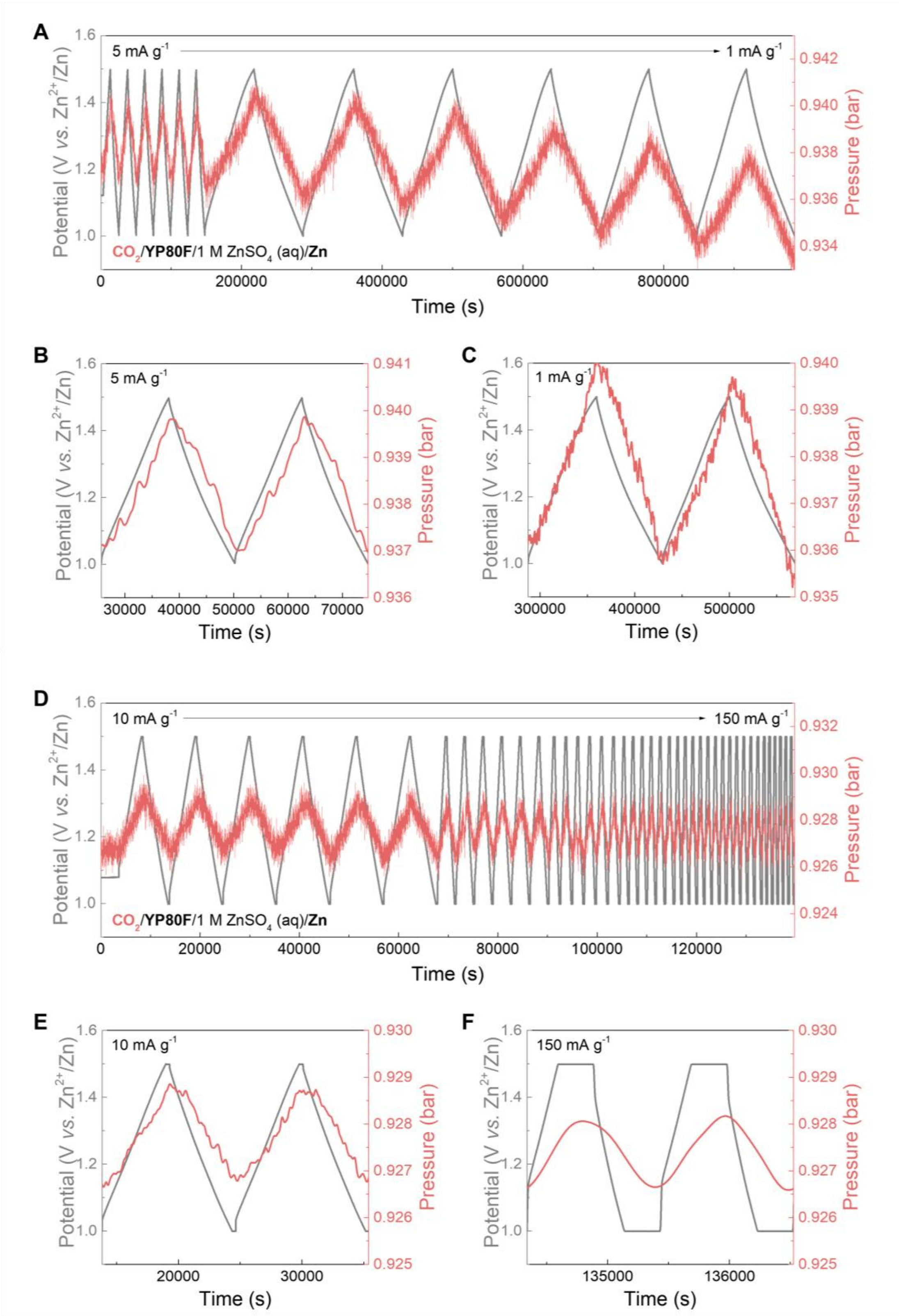

Figure S14. Electrochemical CO_2 capture measurements of the hybrid capacitor under CO_2. (**A**) Overall GCD curves (grey) and corresponding pressure curves (red) of the device with the asymmetric cell configuration of "CO_2/YP80F/1 M $ZnSO_4$ (aq)/Zn" at different current densities from 5 to 1 mA g^{-1} in the positive charging mode, all with 5-min voltage/potential holds. Zoomed GCD curves (grey) and smoothed pressure curves (averaged every 100 sec, red)

of the device with the asymmetric cell configuration of "CO_2/YP80F/1 M $ZnSO_4$ (aq)/Zn" at the current densities of (**B**) 5 mA g^{-1} and (**C**) 1 mA g^{-1} in the positive charging mode, with 5-min voltage/potential holds. (**D**) Overall GCD curves (grey) and corresponding pressure curves (red) of the device with the asymmetric cell configuration of "CO_2/YP80F/1 M $ZnSO_4$ (aq)/Zn" at different current densities from 10 to 150 mA g^{-1} in the positive charging mode, all with 5-min voltage/potential holds. Zoomed GCD curves (grey) and smoothed pressure curves (averaged every 100 sec, red) of the device with the asymmetric cell configuration of "CO_2/YP80F/1 M $ZnSO_4$ (aq)/Zn" at the current densities of (**E**) 10 mA g^{-1} and (**F**) 150 mA g^{-1} in the positive charging mode, with 5-min voltage/potential holds.

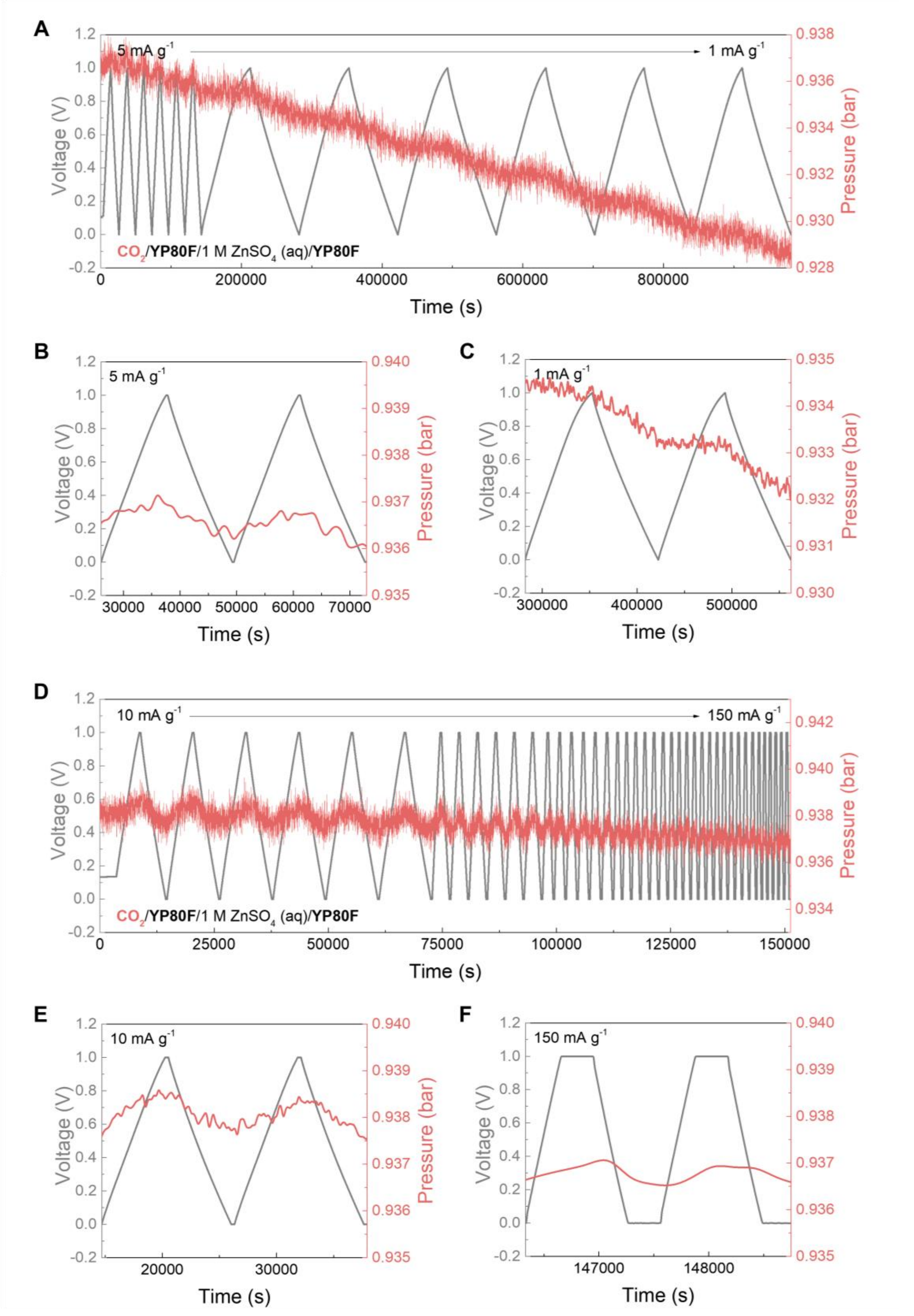

Figure S15. Electrochemical CO_2 capture measurements of the symmetric supercapacitor under CO_2. (**A**) Overall GCD curves (grey) and corresponding pressure curves (red) of the device with the symmetric cell configuration of "CO_2/YP80F/1 M $ZnSO_4$ (aq)/YP80F" at different current densities from 5 to 1 mA g^{-1} in the positive charging mode, all with 5-min voltage/potential holds. Zoomed GCD curves (grey) and smoothed pressure curves (averaged

every 100 sec, red) of the device with the symmetric cell configuration of "CO_2/YP80F/1 M $ZnSO_4$ (aq)/YP80F" at the current densities of (**B**) 5 mA g^{-1} and (**C**) 1 mA g^{-1} in the positive charging mode, with 5-min voltage/potential holds. (**D**) Overall GCD curves (grey) and corresponding pressure curves (red) of the device with the symmetric cell configuration of "CO_2/YP80F/1 M $ZnSO_4$ (aq)/YP80F" at different current densities from 10 to 150 mA g^{-1} in the positive charging mode, all with 5-min voltage/potential holds. Zoomed GCD curves (grey) and smoothed pressure curves (averaged every 100 sec, red) of the device with the symmetric cell configuration of "CO_2/YP80F/1 M $ZnSO_4$ (aq)/YP80F" at the current densities of (**E**) 10 mA g^{-1} and (**F**) 150 mA g^{-1} in the positive charging mode, with 5-min voltage/potential holds. Notes: The irreversible pressure decrease observed at the slow charging conditions is likely due to corrosion processes, similar to what we saw for the hybrid capacitor in the negative charging mode.

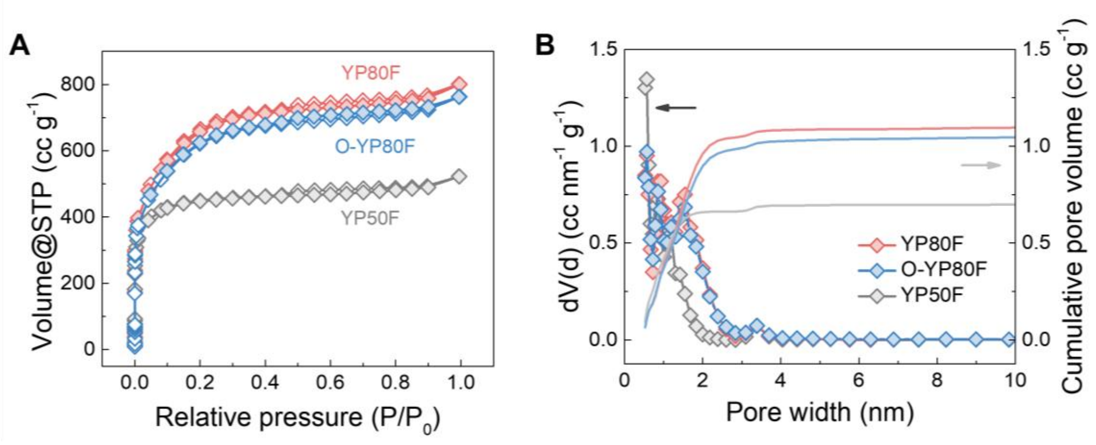

Figure S16. Pore structure analysis of YP series of activated porous carbons. (**A**) N_2 sorption isotherms at 77 K of YP80F, YP50F and O-YP80F. (**B**) Pore size distribution and cumulative pore volume using the quenched solid density functional theory (QSDFT) and slit pore model, of YP80F, O-YP80F and YP50F.

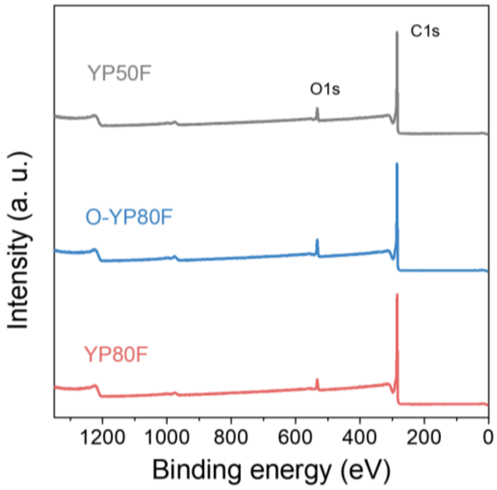

Figure S17. Surface chemistry analysis of YP series of activated porous carbons. XPS survey of YP80F, O-YP80F and YP50F.

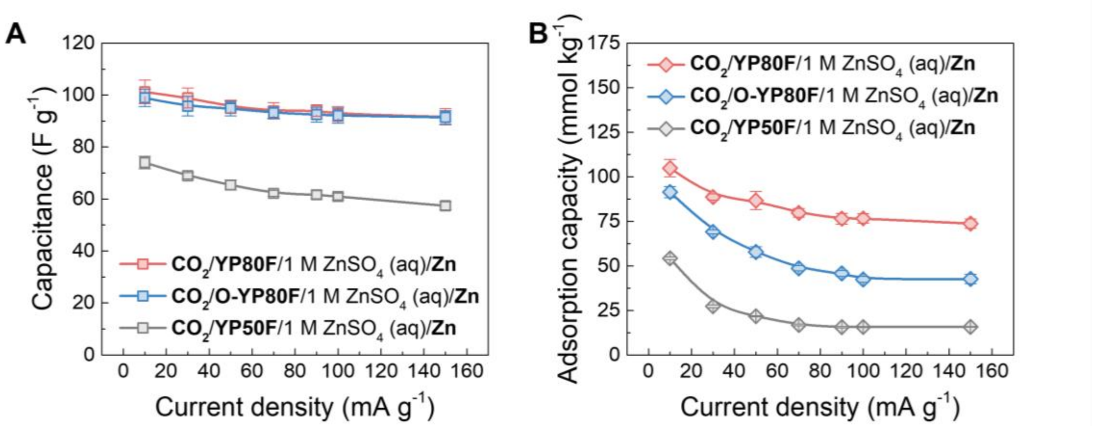

Figure S18. The effects of activated carbon electrode materials on the performance of hybrid capacitors under CO_2. Comparison of (**A**) the discharge capacitances and (**B**) CO_2 adsorption capacities of the devices with the asymmetric cell configurations of "CO_2/YP80F/1 M $ZnSO_4$ (aq)/Zn", "CO_2/O-YP80F/1 M $ZnSO_4$ (aq)/Zn" and "CO_2/YP50F/1 M $ZnSO_4$ (aq)/Zn" at different current densities from 10 to 150 mA g^{-1} in the positive charging mode, with 5-min voltage/potential holds. All the discharge capacitances and CO_2 adsorption capacities were normalized based on the active mass of the working electrode. The error was calculated using a 95% confidence interval with the Student's t-test.

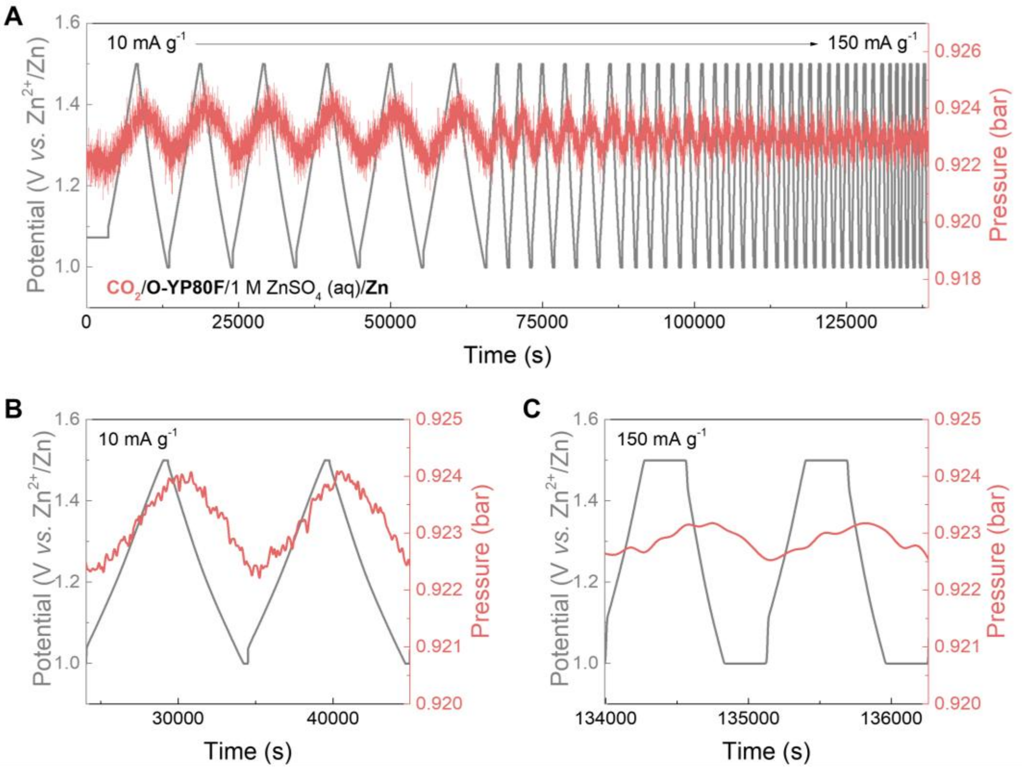

Figure S19. Electrochemical CO_2 capture measurements of the hybrid capacitor under CO_2. (**A**) Overall GCD curves (grey) and corresponding pressure curves (red) of the device with the asymmetric cell configuration of "CO_2/O-YP80F/1 M $ZnSO_4$ (aq)/Zn" at different current densities from 10 to 150 mA g^{-1} in the positive charging mode, all with 5-min voltage/potential holds. Zoomed GCD curves (grey) and smoothed pressure curves (averaged every 100 sec, red) of the device with the asymmetric cell configuration of "CO_2/O-YP80F/1 M $ZnSO_4$ (aq)/Zn" at the current densities of (**B**) 10 mA g^{-1} and (**C**) 150 mA g^{-1} in the positive charging mode, with 5-min voltage/potential holds.

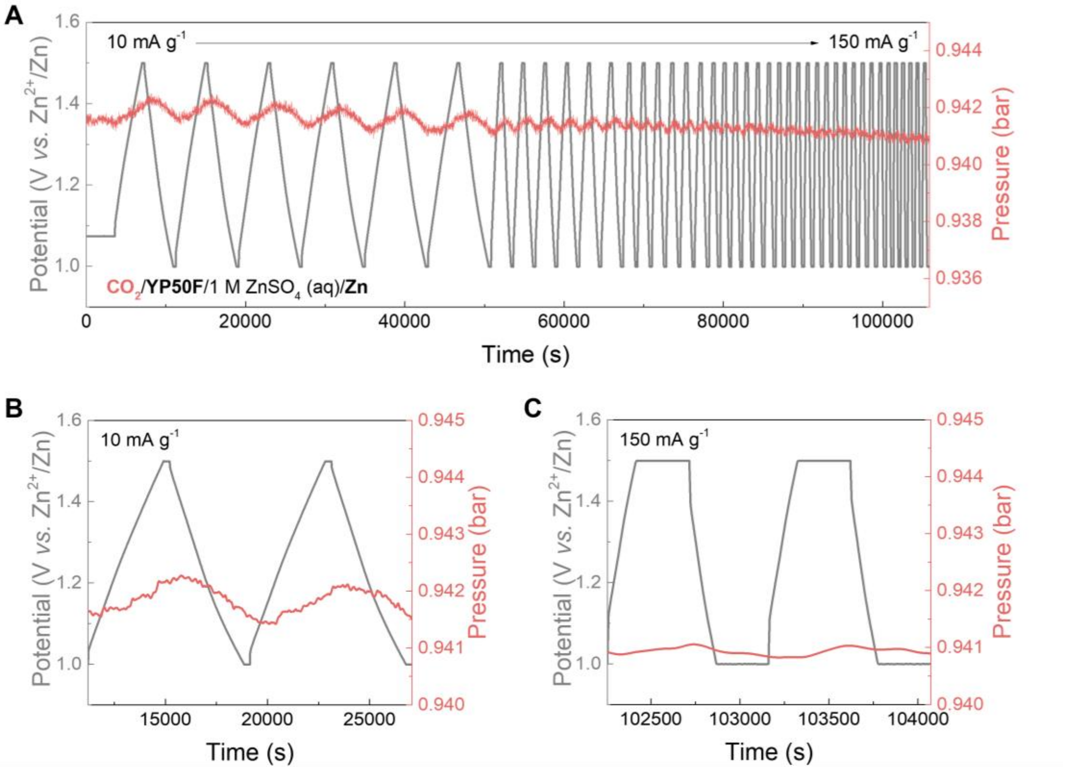

Figure S20. Electrochemical CO_2 capture measurements of the hybrid capacitor under CO_2. (**A**) Overall GCD curves (grey) and corresponding pressure curves (red) of the device with the asymmetric cell configuration of "CO_2/YP50F/1 M $ZnSO_4$ (aq)/Zn" at different current densities from 10 to 150 mA g^{-1} in the positive charging mode, all with 5-min voltage/potential holds. Zoomed GCD curves (grey) and smoothed pressure curves (averaged every 100 sec, red) of the device with the asymmetric cell configuration of "CO_2/YP50F/1 M $ZnSO_4$ (aq)/Zn" at the current densities of (**B**) 10 mA g^{-1} and (**C**) 150 mA g^{-1} in the positive charging mode, with 5-min voltage/potential holds.

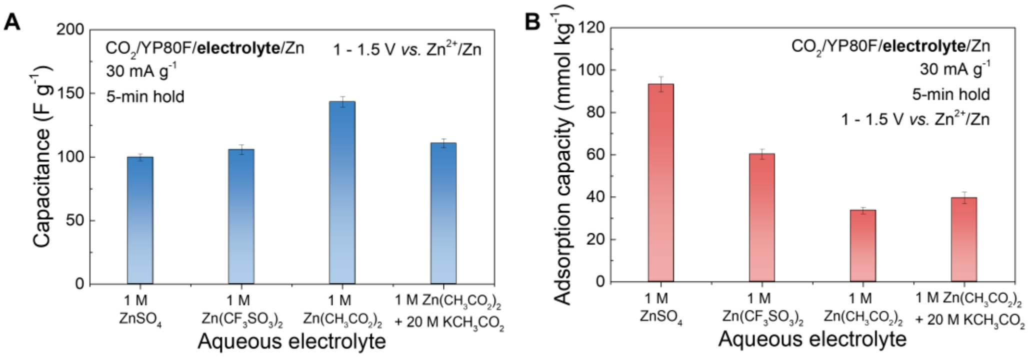

Figure S21. The effects of electrolyte compositions on the performance of hybrid capacitors under CO_2. Comparison of (**A**) the discharge capacitances and (**B**) CO_2 adsorption capacities of the devices with the asymmetric cell configurations of "CO_2/YP80F/1 M $ZnSO_4$ (aq)/Zn", "CO_2/YP80F/1 M $Zn(CF_3SO_3)_2$ (aq)/Zn", "CO_2/YP80F/1 M $Zn(CH_3CO_2)_2$ (aq)/Zn", and "CO_2/YP80F/1 M $Zn(CH_3CO_2)_2$ + 20 M KCH_3CO_2 (aq)/Zn" at the current density of 30 mA g^{-1} in the positive charging mode, with 5-min voltage/potential holds. All the discharge capacitances and CO_2 adsorption capacities were normalized based on the active mass of the working electrode. The error was calculated using a 95% confidence interval with the Student's t-test.

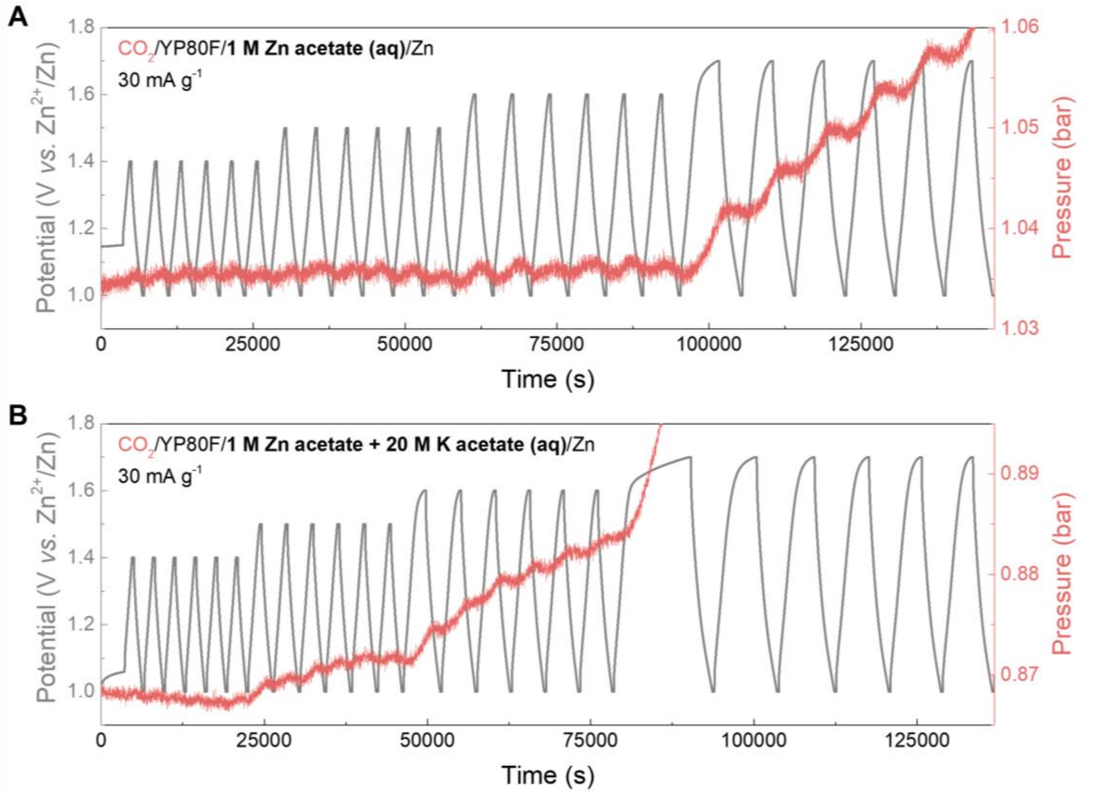

Figure S22. Exploring the upper potential limitation of hybrid capacitors under CO_2. Overall GCD curves (grey) and corresponding pressure curves (red) of the devices with the asymmetric cell configurations of (**A**) "CO_2/YP80F/1 M $Zn(CH_3CO_2)_2$ (aq)/Zn" and (**B**) "CO_2/YP80F/1 M $Zn(CH_3CO_2)_2$ + 20 M KCH_3CO_2 (aq)/Zn" at the current density of 30 mA g^{-1} in different positive charging modes, all with 5-min voltage/potential holds.

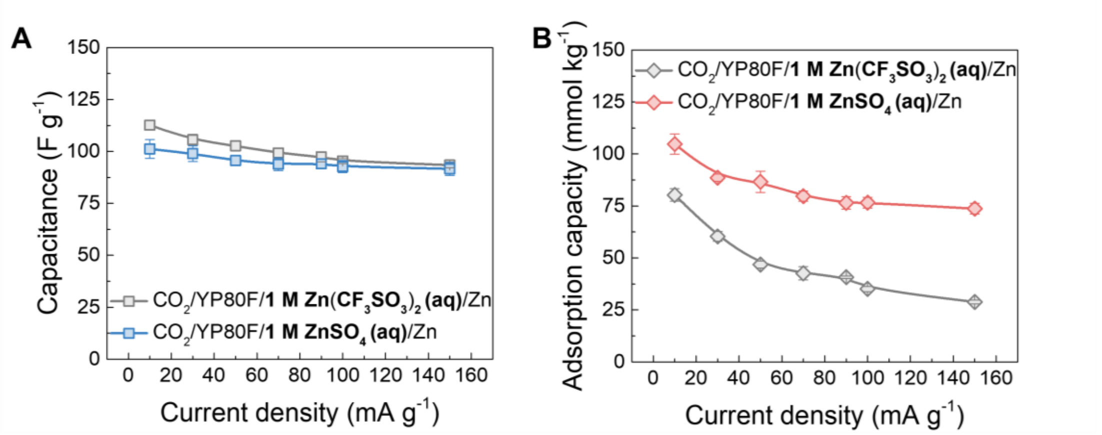

Figure S23. The effects of electrolyte compositions on the performance of hybrid capacitors under CO_2. Comparison of (**A**) the discharge capacitances and (**B**) CO_2 adsorption capacities of the devices with the asymmetric cell configurations of "CO_2/YP80F/1 M $ZnSO_4$ (aq)/Zn" and "CO_2/YP80F/1 M $Zn(CF_3SO_3)_2$ (aq)/Zn" at different current densities from 10 to 150 mA g^{-1} in the positive charging mode, with 5-min voltage/potential holds. All the discharge capacitances and CO_2 adsorption capacities are normalized based on the active mass of the working electrode. The error was calculated using a 95% confidence interval with the Student's t-test.

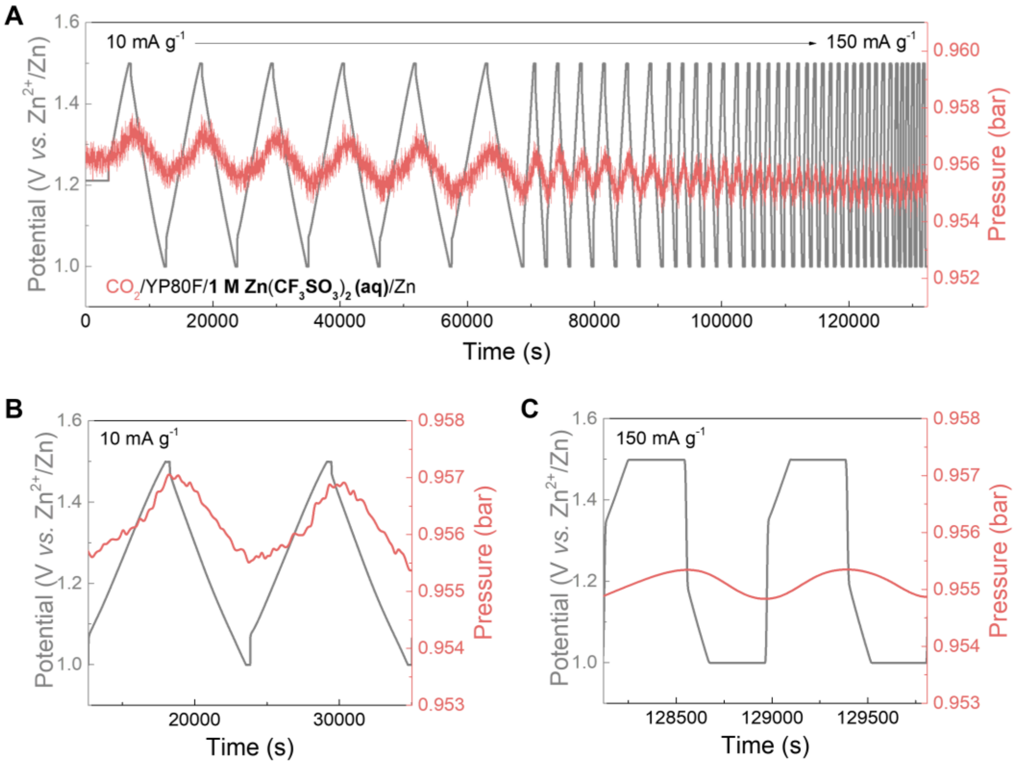

Figure S24. Electrochemical CO_2 capture measurements of the hybrid capacitor under CO_2. (**A**) Overall GCD curves (grey) and corresponding pressure curves (red) of the device with the asymmetric cell configuration of "CO_2/YP80F/1 M $Zn(CF_3SO_3)_2$ (aq)/Zn" at different current densities from 10 to 150 mA g^{-1} in the positive charging mode, all with 5-min voltage/potential holds. Zoomed GCD curves (grey) and smoothed pressure curves (averaged every 100 sec, red) of the device with the asymmetric cell configuration of "CO_2/YP80F/1 M $Zn(CF_3SO_3)_2$ (aq)/Zn" at the current densities of (**B**) 10 mA g^{-1} and (**C**) 150 mA g^{-1} in the positive charging mode, with 5-min voltage/potential holds.

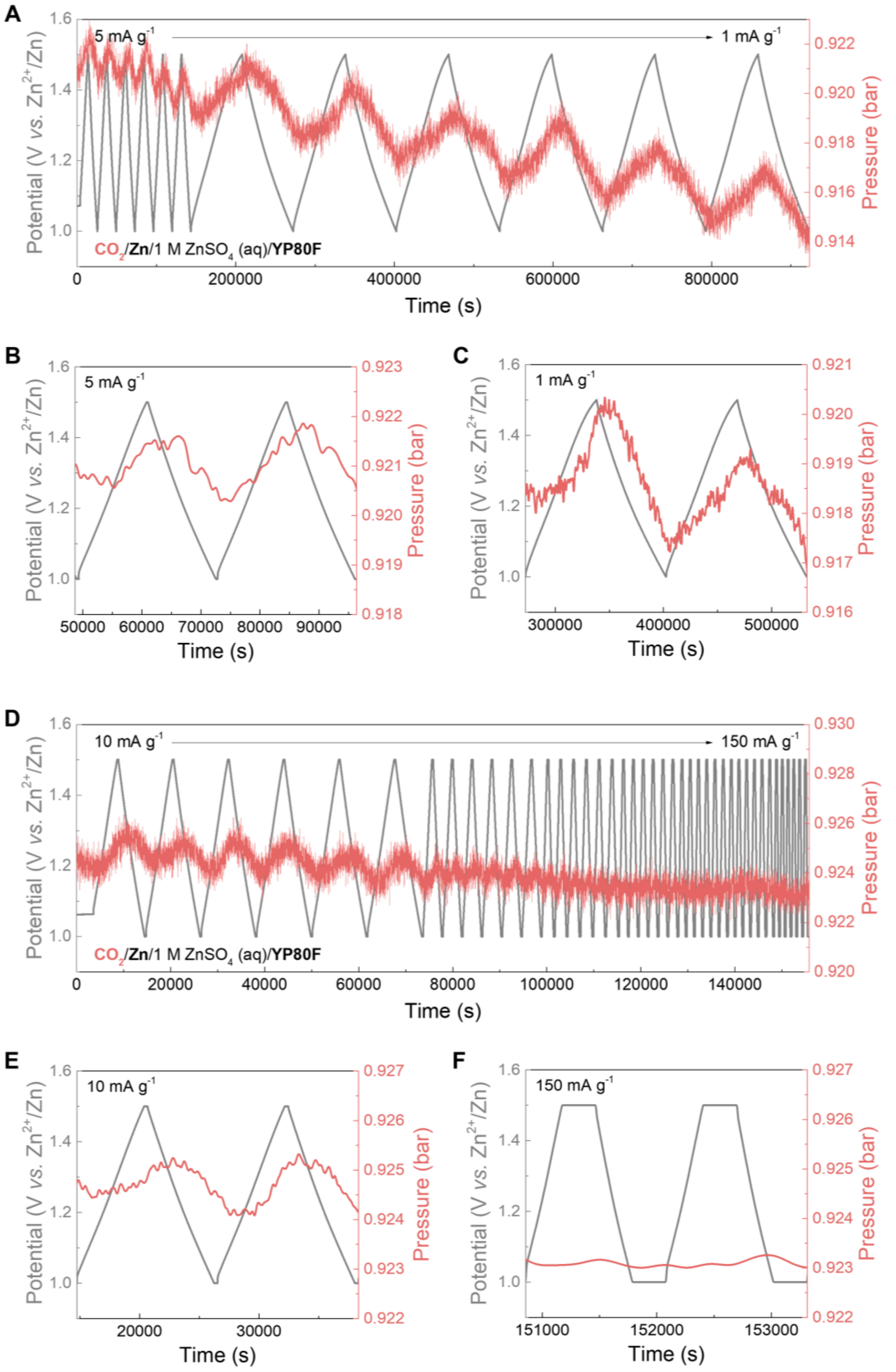

Figure S25. Electrochemical CO_2 capture measurements of the hybrid capacitor with the flipped configuration under CO_2. (**A**) Overall GCD curves (grey) and corresponding pressure curves (red) of the device with the asymmetric cell configuration of "CO_2/Zn/1 M $ZnSO_4$ (aq)/YP80F" at different current densities from 5 to 1 mA g^{-1} in the positive charging mode, all

with 5-min voltage/potential holds. Zoomed GCD curves (grey) and smoothed pressure curves (averaged every 100 sec, red) of the device with the asymmetric cell configuration of "CO_2/Zn/1 M $ZnSO_4$ (aq)/YP80F" at the current densities of (**B**) 5 mA g^{-1} and (**C**) 1 mA g^{-1} in the positive charging mode, with 5-min voltage/potential holds. (**D**) Overall GCD curves (grey) and corresponding pressure curves (red) of the device with the asymmetric cell configuration of "CO_2/Zn/1 M $ZnSO_4$ (aq)/YP80F" at different current densities from 10 to 150 mA g^{-1} in the positive charging mode, all with 5-min voltage/potential holds. Zoomed GCD curves (grey) and smoothed pressure curves (averaged every 100 sec, red) of the device with the asymmetric cell configuration of "CO_2/Zn/1 M $ZnSO_4$ (aq)/YP80F" at the current densities of (**E**) 10 mA g^{-1} and (**F**) 150 mA g^{-1} in the positive charging mode, with 5-min voltage/potential holds. Notes: Here, when connected to the potentiostat, the open-circuit potential of the hybrid capacitor was around 1 V *vs.* Zn^{2+}/Zn. When the potential changed from 1 to 1.5 V *vs.* Zn^{2+}/Zn, the electrolyte-immersed YP80F electrode carried positive charges, namely still in the positive charging mode. We still saw the CO_2 capture when the electrolyte-immersed YP80F electrode obtained electrons.

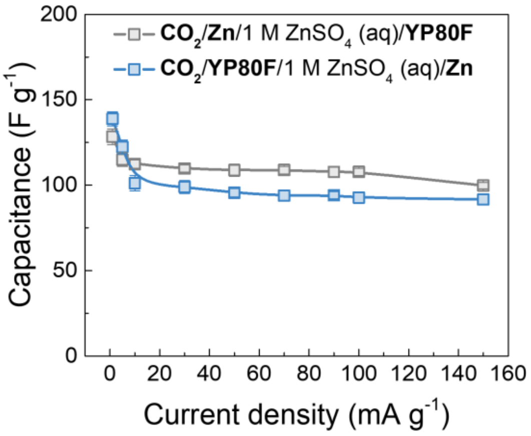

Figure S26. Impact of immersion of the carbon electrode on electrochemical CO_2 capture performance. Comparison of the discharge capacitances of the hybrid capacitor with a normal configuration of "CO_2/YP80F/1 M $ZnSO_4$ (aq)/Zn" and that with a flipped configuration of "CO_2/Zn/1 M $ZnSO_4$ (aq)/YP80F" under CO_2 at different current densities from 1 to 150 mA g^{-1} in the positive charging mode, with 5-min voltage/potential holds. All the discharge capacitances were normalized based on the active mass of the working electrode. The error was calculated using a 95% confidence interval with the Student's t-test. Notes: With the flipped cell configuration noted as "CO_2/Zn/1 M $ZnSO_4$ (aq)/YP80F", the electrochemical capacitances of the hybrid capacitor increased by ~20% at fast charging conditions (*e.g.,* increasing from 92 to 108 F g^{-1} at 100 mA g^{-1}), which indicates the improved charge storage kinetics due to the better wettability of the carbon electrode.

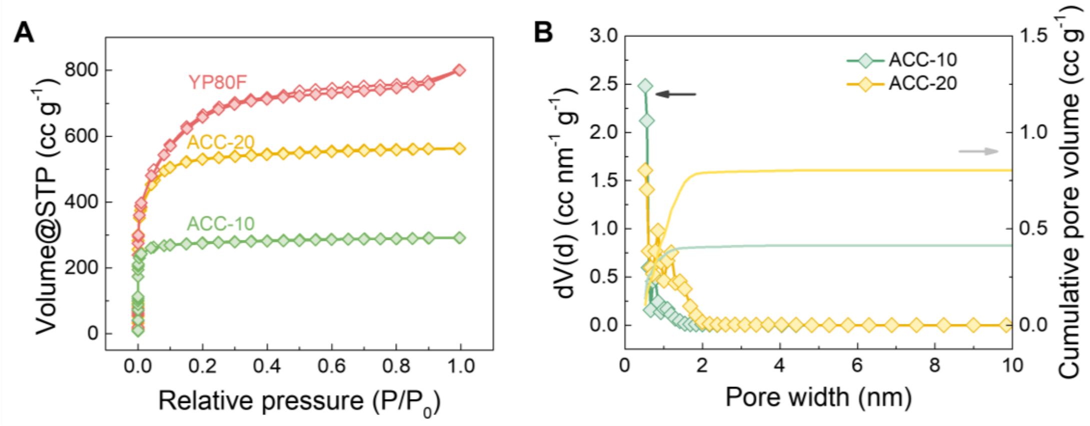

Figure S27. Pore structure analysis of ACC series of activated microporous carbons. (**A**) N_2 sorption isotherms at 77 K of ACC-20 and ACC-10 compared to YP80F. (**B**) Pore size distribution and cumulative pore volume using the quenched solid density functional theory (QSDFT) and slit pore model, of ACC-20 and ACC-10.

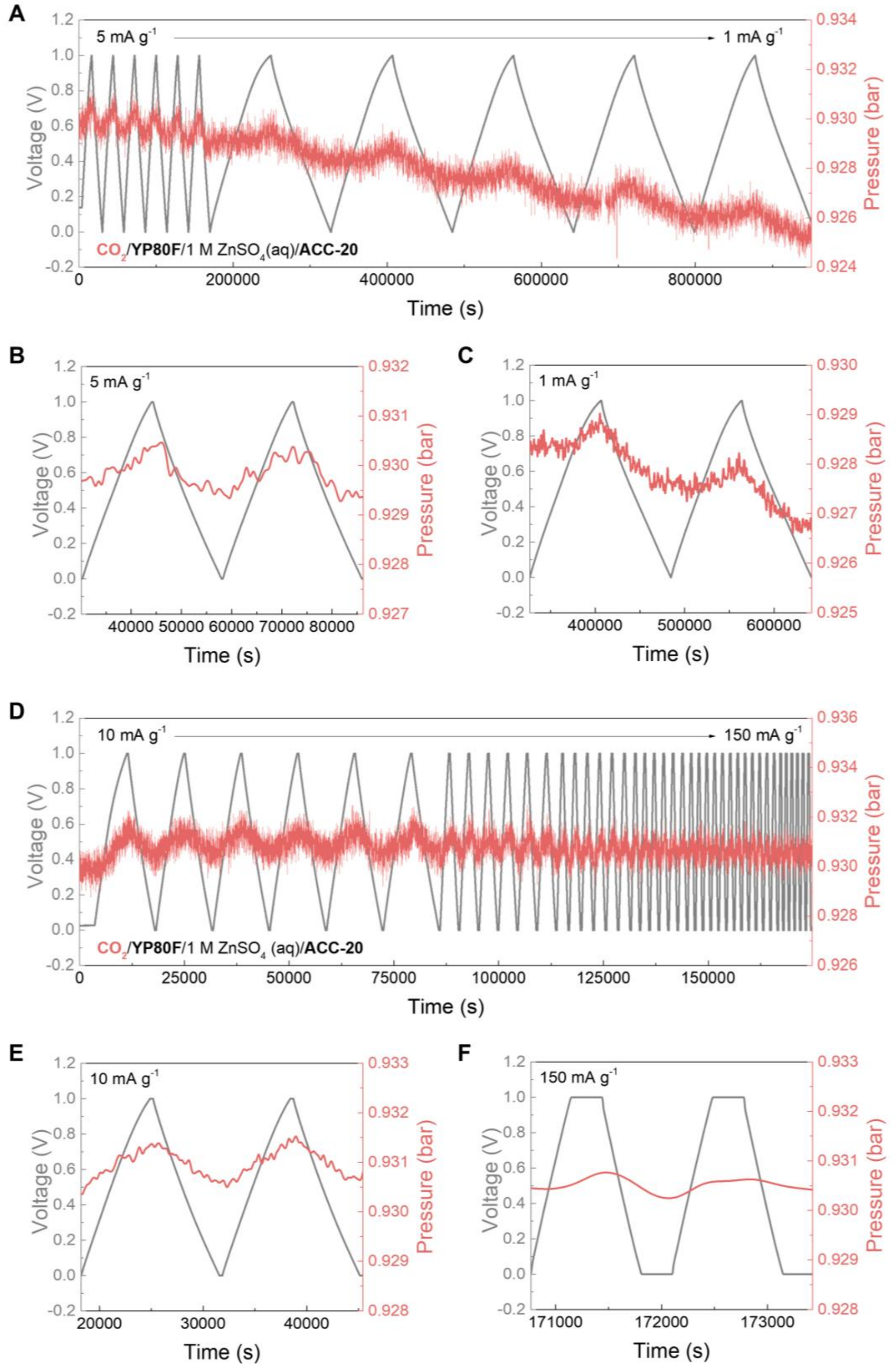

Figure S28. Electrochemical CO_2 capture measurements of the asymmetric supercapacitor under CO_2. (**A**) Overall GCD curves (grey) and corresponding pressure curves (red) of the device with the asymmetric cell configuration of "CO_2/YP80F/1 M $ZnSO_4$ (aq)/ACC-20" at different current densities from 5 to 1 mA g^{-1} in the positive charging mode, all with 5-min voltage/potential holds. Zoomed GCD curves (grey) and smoothed pressure

curves (averaged every 100 sec, red) of the device with the asymmetric cell configuration of "CO_2/YP80F/1 M $ZnSO_4$ (aq)/ACC-20" at the current densities of (**B**) 5 mA g^{-1} and (**C**) 1 mA g^{-1} in the positive charging mode, with 5-min voltage/potential holds. (**D**) Overall GCD curves (grey) and corresponding pressure curves (red) of the device with the asymmetric cell configuration of "CO_2/YP80F/1 M $ZnSO_4$ (aq)/ACC-20" at different current densities from 10 to 150 mA g^{-1} in the positive charging mode, all with 5-min voltage/potential holds. Zoomed GCD curves (grey) and smoothed pressure curves (averaged every 100 sec, red) of the device with the asymmetric cell configuration of "CO_2/YP80F/1 M $ZnSO_4$ (aq)/ACC-20" at the current densities of (**E**) 10 mA g^{-1} and (**F**) 150 mA g^{-1} in the positive charging mode, with 5-min voltage/potential holds. Notes: Here the active mass ratio of YP80F to ACC-20 was adjusted to 1.1 to balance the charges and equally allocate the voltages. Their individual electrochemical capacitances were evaluated in our previous study.[8]

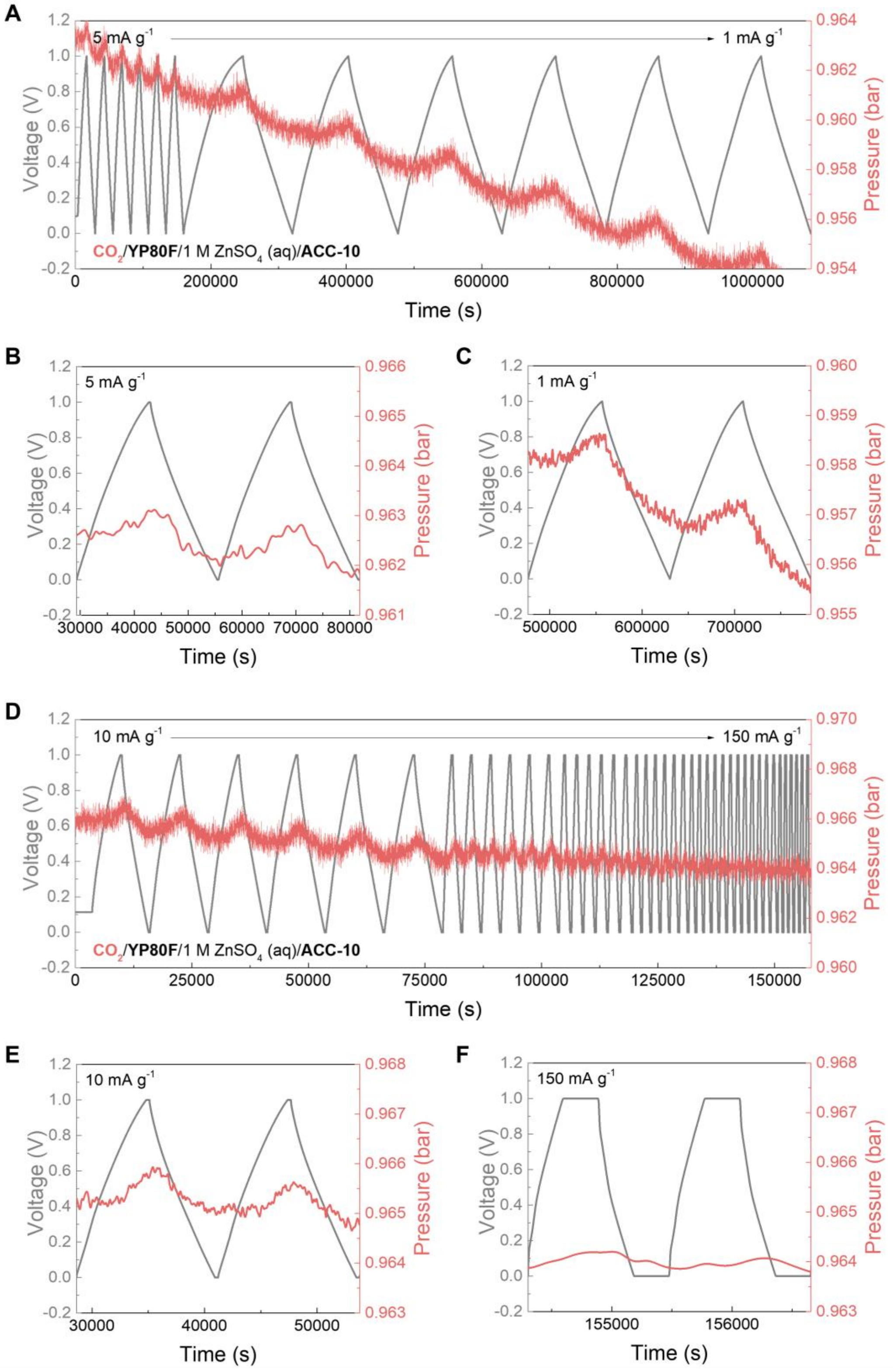

Figure S29. Electrochemical CO_2 capture measurements of the asymmetric supercapacitor under CO_2. (**A**) Overall GCD curves (grey) and corresponding pressure curves (red) of the device with the asymmetric cell configuration of "CO_2/YP80F/1 M $ZnSO_4$ (aq)/ACC-10" at different current densities from 5 to 1 mA g^{-1} in the positive charging mode, all with 5-min voltage/potential holds. Zoomed GCD curves (grey) and smoothed pressure

curves (averaged every 100 sec, red) of the device with the asymmetric cell configuration of "CO_2/YP80F/1 M $ZnSO_4$ (aq)/ACC-10" at the current densities of (**B**) 5 mA g^{-1} and (**C**) 1 mA g^{-1} in the positive charging mode, with 5-min voltage/potential holds. (**D**) Overall GCD curves (grey) and corresponding pressure curves (red) of the device with the asymmetric cell configuration of "CO_2/YP80F/1 M $ZnSO_4$ (aq)/ACC-10" at different current densities from 10 to 150 mA g^{-1} in the positive charging mode, all with 5-min voltage/potential holds. Zoomed GCD curves (grey) and smoothed pressure curves (averaged every 100 sec, red) of the device with the asymmetric cell configuration of "CO_2/YP80F/1 M $ZnSO_4$ (aq)/ACC-20" at the current densities of (**E**) 10 mA g^{-1} and (**F**) 150 mA g^{-1} in the positive charging mode, with 5-min voltage/potential holds. Notes: Here the active mass ratio of YP80F to ACC-10 was adjusted to 1.3 to balance the charges and equally allocate the voltages. Their individual electrochemical capacitances were evaluated in our previous study.[8]

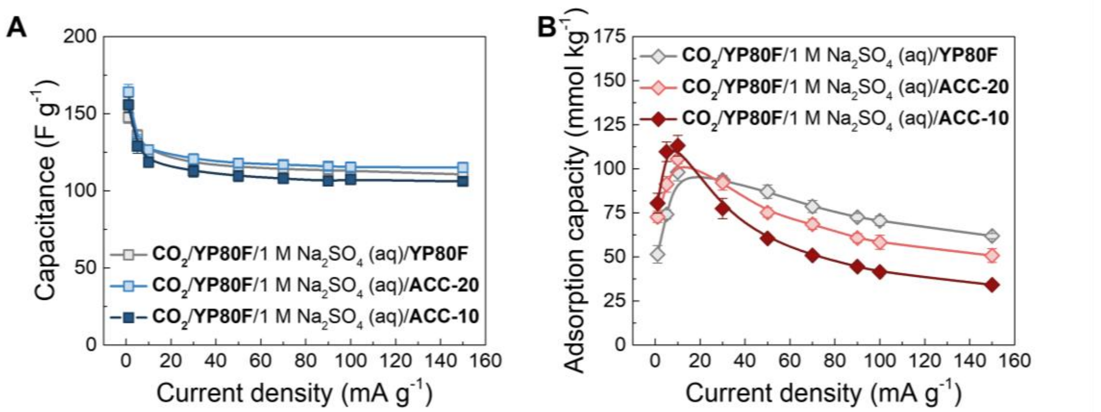

Figure S30. The effects of electrolyte types on the performance of asymmetric supercapacitors under CO_2. Comparison of (**A**) the discharge capacitances and (**B**) CO_2 adsorption capacities of the devices with the symmetric cell configuration of "CO_2/YP80F/1 M Na_2SO_4 (aq)/YP80F" and the asymmetric cell configuration of "CO_2/YP80F/1 M Na_2SO_4 (aq)/ACC-20" and "CO_2/YP80F/1 M Na_2SO_4 (aq)/ACC-10" at different current densities from 1 to 150 mA g^{-1} in the positive charging mode, with 5-min voltage/potential holds. All the discharge capacitances and CO_2 adsorption capacities were normalized based on the active mass of the working electrode. The error was calculated using a 95% confidence interval with the Student's t-test.

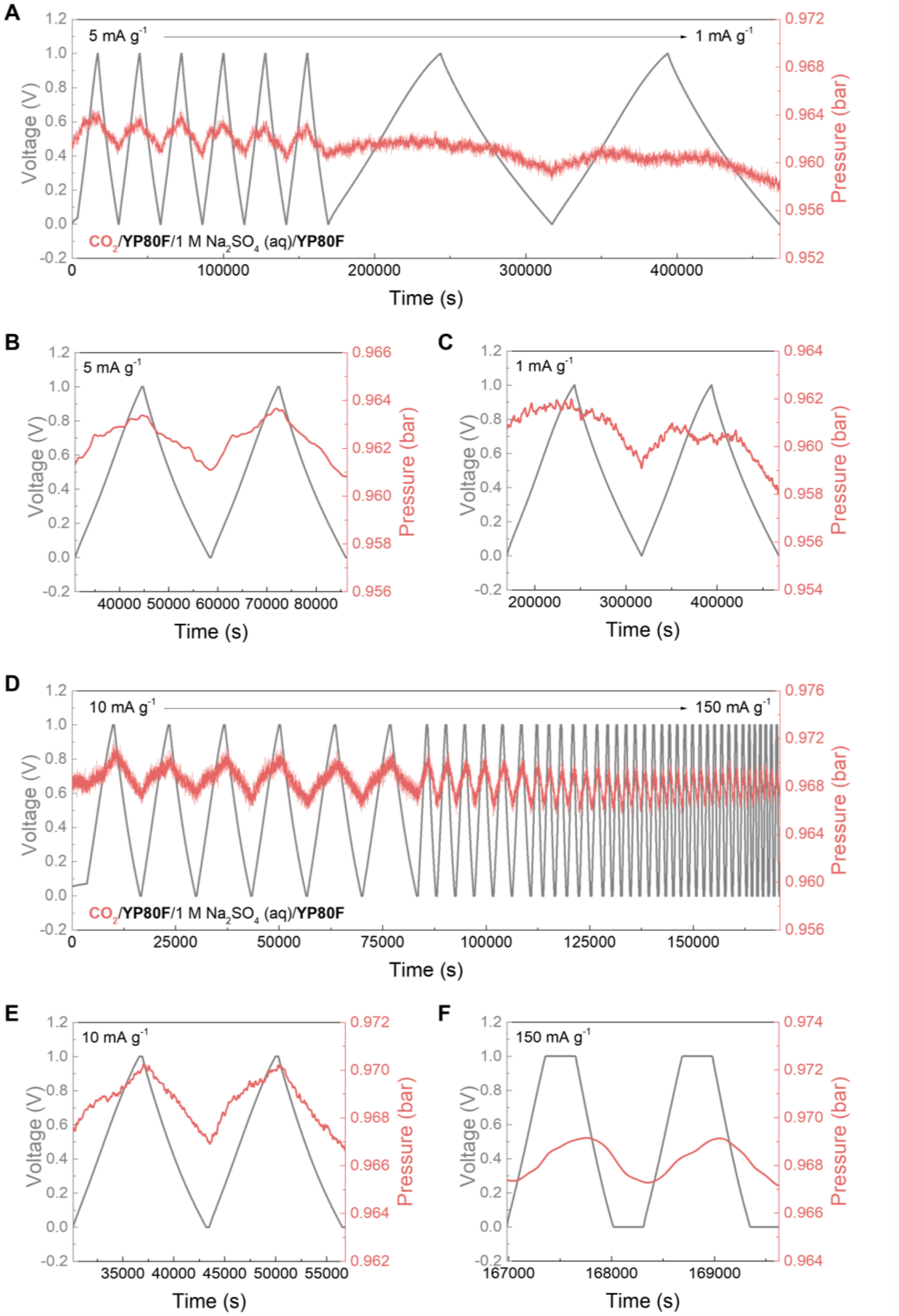

Figure S31. Electrochemical CO_2 capture measurements of the asymmetric supercapacitor under CO_2. (**A**) Overall GCD curves (grey) and corresponding pressure curves (red) of the device with the symmetric cell configuration of "CO_2/YP80F/1 M Na_2SO_4 (aq)/YP80F" at different current densities from 5 to 1 mA g^{-1} in the positive charging mode, all with 5-min voltage/potential holds. Zoomed GCD curves (grey) and smoothed pressure curves

(averaged every 100 sec, red) of the device with the symmetric cell configuration of "CO_2/YP80F/1 M Na_2SO_4 (aq)/YP80F" at the current densities of (**B**) 5 mA g^{-1} and (**C**) 1 mA g^{-1} in the positive charging mode, with 5-min voltage/potential holds. (**D**) Overall GCD curves (grey) and corresponding pressure curves (red) of the device with the symmetric cell configuration of "CO_2/YP80F/1 M Na_2SO_4 (aq)/YP80F" at different current densities from 10 to 150 mA g^{-1} in the positive charging mode, all with 5-min voltage/potential holds. Zoomed GCD curves (grey) and smoothed pressure curves (averaged every 100 sec, red) of the device with the symmetric cell configuration of "CO_2/YP80F/1 M Na_2SO_4 (aq)/YP80F" at the current densities of (**E**) 10 mA g^{-1} and (**F**) 150 mA g^{-1} in the positive charging mode, with 5-min voltage/potential holds.

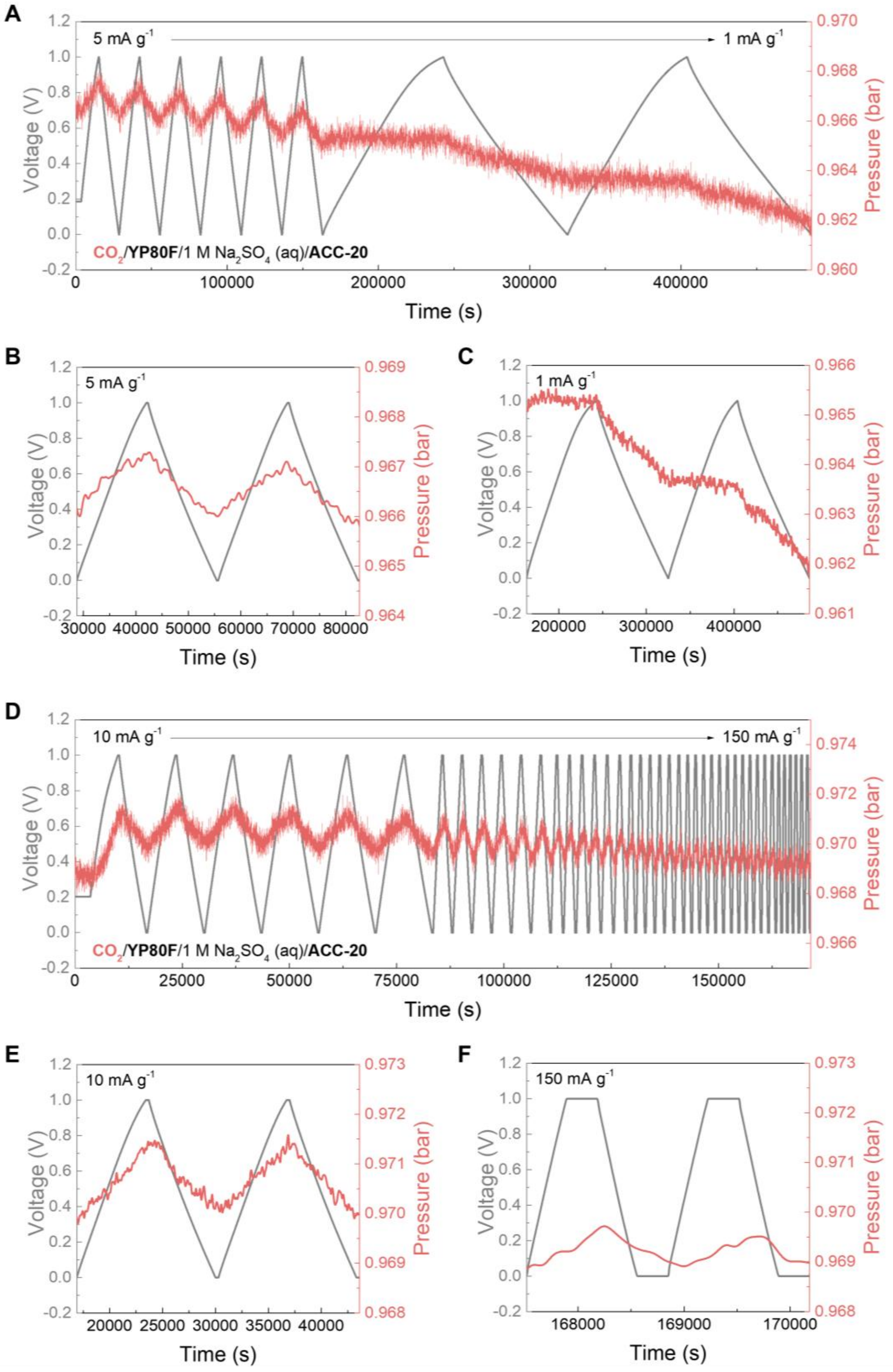

Figure S32. Electrochemical CO_2 capture measurements of the asymmetric supercapacitor under CO_2. (**A**) Overall GCD curves (grey) and corresponding pressure curves (red) of the device with the asymmetric cell configuration of "CO_2/YP80F/1 M Na_2SO_4 (aq)/ACC-20" at different current densities from 5 to 1 mA g^{-1} in the positive charging mode, all with 5-min voltage/potential holds. Zoomed GCD curves (grey) and smoothed pressure

curves (averaged every 100 sec, red) of the device with the asymmetric cell configuration of "CO_2/YP80F/1 M Na_2SO_4 (aq)/ACC-20" at the current densities of (**B**) 5 mA g^{-1} and (**C**) 1 mA g^{-1} in the positive charging mode, with 5-min voltage/potential holds. (**D**) Overall GCD curves (grey) and corresponding pressure curves (red) of the device with the asymmetric cell configuration of "CO_2/YP80F/1 M Na_2SO_4 (aq)/ACC-20" at different current densities from 10 to 150 mA g^{-1} in the positive charging mode, all with 5-min voltage/potential holds. Zoomed GCD curves (grey) and smoothed pressure curves (averaged every 100 sec, red) of the device with the asymmetric cell configuration of "CO_2/YP80F/1 M Na_2SO_4 (aq)/ACC-20" at the current densities of (**E**) 10 mA g^{-1} and (**F**) 150 mA g^{-1} in the positive charging mode, with 5-min voltage/potential holds. Notes: Here the active mass ratio of YP80F to ACC-20 was adjusted to 1.1 to balance the charges and equally allocate the voltages. Their individual electrochemical capacitances were evaluated in our previous study.[8]

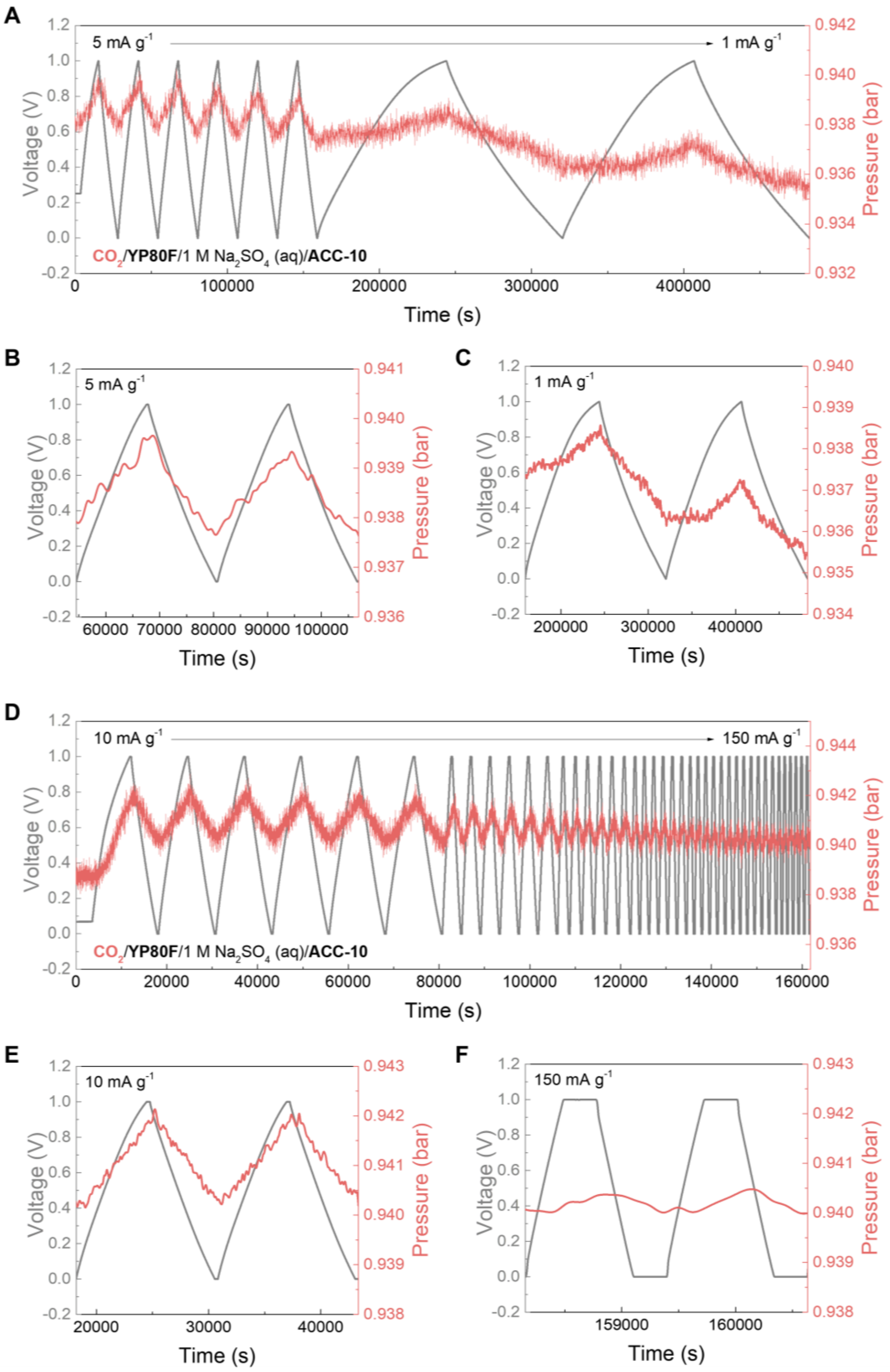

Figure S33. Electrochemical CO_2 capture measurements of the asymmetric supercapacitor under CO_2. (**A**) Overall GCD curves (grey) and corresponding pressure curves (red) of the device with the asymmetric cell configuration of "CO_2/YP80F/1 M Na_2SO_4 (aq)/ACC-10" at different current densities from 5 to 1 mA g^{-1} in the positive charging mode, all with 5-min voltage/potential holds. Zoomed GCD curves (grey) and smoothed pressure

curves (averaged every 100 sec, red) of the device with the asymmetric cell configuration of "CO_2/YP80F/1 M Na_2SO_4 (aq)/ACC-10" at the current densities of (**B**) 5 mA g^{-1} and (**C**) 1 mA g^{-1} in the positive charging mode, with 5-min voltage/potential holds. (**D**) Overall GCD curves (grey) and corresponding pressure curves (red) of the device with the asymmetric cell configuration of "CO_2/YP80F/1 M Na_2SO_4 (aq)/ACC-10" at different current densities from 10 to 150 mA g^{-1} in the positive charging mode, all with 5-min voltage/potential holds. Zoomed GCD curves (grey) and smoothed pressure curves (averaged every 100 sec, red) of the device with the asymmetric cell configuration of "CO_2/YP80F/1 M Na_2SO_4 (aq)/ACC-10" at the current densities of (**E**) 10 mA g^{-1} and (**F**) 150 mA g^{-1} in the positive charging mode, with 5-min voltage/potential holds. Notes: Here the active mass ratio of YP80F to ACC-10 was adjusted to 1.3 to balance the charges and equally allocate the voltages. Their individual electrochemical capacitances were evaluated in our previous study.[8]

Table S1. Comparison between the ionic charge capacity derived from CO_2 capture and the electrochemical discharge capacity of the working electrode in the hybrid capacitor.

**Current ($mA\ g^{-1}$)**	**1**	**5**	**10**	**30**	**50**	**70**	**90**	**100**	**150**
$\mathbf{Q_{HCO3-}}$ ($C\ g^{-1}$)	20.0	13.4	10.1	8.5	8.4	7.7	7.4	7.4	7.1
$\mathbf{Q_{total}}$ ($C\ g^{-1}$)	69.4	61.2	50.6	49.5	47.8	46.9	47.0	46.3	45.8

Notes: Q_{HCO3-} is the ionic charge capacity derived from CO_2 capture where we assume one captured CO_2 is related to one HCO_3^- ion, and Q_{total} is the electrochemical discharge capacity of the working electrode in the hybrid capacitor.

Table S2. Pore structure details of carbon electrodes.

	S_{BET}[a] (m^2 g^{-1})	V_t[b] (cm^3 g^{-1})	D_A[c] (nm)
**YP50F**	1694	0.73	0.87
**YP80F**	2324	1.14	1.13
**O-YP80F**	2243	1.09	1.18
**ACC-10**	1094	0.43	0.51
**ACC-20**	2004	0.82	0.88

Notes: [a]Brunauer–Emmett–Teller (BET) specific surface area; [b]Total pore volume; [c]average pore diameter.

Table S3. Functional group details of carbon electrodes.

	C (at%)	O (at%)	Si (at%)
**YP50F**	93.51 ± 0.28	**6.49** ± 0.02	N/A
**YP80F**	94.53 ± 0.28	**5.14** ± 0.04	N/A
**O-YP80F**	91.81 ± 0.19	**8.13** ± 0.01	N/A
**ACC-10**	78.04 ± 0.35	13.96 ± 0.09 (**6.40**)	8.20 ± 0.03
**ACC-20**	86.43 ± 1.58	9.77 ± 0.15 (**6.34**)	3.80 ± 0.02

Notes: Oxygen amount in the ( ) is the O-C amount, excluding the O-Si amount using the fitting of O1s spectra; SiO_2 was added as reinforcement particles in the commercial free-standing carbon electrodes, which is electrochemically inactive in the aqueous electrolyte. Error bars represent the standard deviation of atomic percentages of elements at different spots on the same sample. N/A (not available).

References

(1) Gor, G. Y.; Thommes, M.; Cychosz, K. A.; Neimark, A. V. Quenched solid density functional theory method for characterization of mesoporous carbons by nitrogen adsorption. *Carbon* **2012**, *50*, 1583-1590.

(2) Morcombe, C. R.; Zilm, K. W. Chemical shift referencing in MAS solid state NMR. *J. Magn. Reson.* **2003**, *162*, 479-486.

(3) Binford, T. B.; Mapstone, G.; Temprano, I.; Forse, A. C. Enhancing the capacity of supercapacitive swing adsorption CO2 capture by tuning charging protocols. *Nanoscale* **2022**, *14*, 7980-7984.

(4) Xu, Z.; Xie, F.; Wang, J.; Au, H.; Tebyetekerwa, M.; Guo, Z.; Yang, S.; Hu, Y. S.; Titirici, M. M. All-Cellulose-Based Quasi-Solid-State Sodium-Ion Hybrid Capacitors Enabled by Structural Hierarchy. *Adv. Funct. Mater.* **2019**, *29*, 1903895.

(5) Zhu, S.; Li, J.; Toth, A.; Landskron, K. Relationships between Electrolyte Concentration and the Supercapacitive Swing Adsorption of CO2. *ACS Appl. Mater. Interfaces* **2019**, *11*, 21489-21495.

(6) Zhu, S.; Li, J.; Toth, A.; Landskron, K. Relationships between the Elemental Composition of Electrolytes and the Supercapacitive Swing Adsorption of CO2. *ACS Appl. Energy Mater.* **2019**, *2*, 7449-7456.

(7) Merlet, C.; Forse, A. C.; Griffin, J. M.; Frenkel, D.; Grey, C. P. Lattice simulation method to model diffusion and NMR spectra in porous materials. *J. Chem. Phys.* **2015**, *142*, 094701.

(8) Xu, Z.; Mapstone, G.; Coady, Z.; Wang, M.; Spreng, T.; Liu, X.; Molino, D.; Forse, A. C. Enhancing electrochemical carbon dioxide capture with supercapacitors. *Nat. Commun.* **2024**, *15*, 7851.

(9) Forse, A. C.; Griffin, J. M.; Presser, V.; Gogotsi, Y.; Grey, C. P. Ring Current Effects: Factors Affecting the NMR Chemical Shift of Molecules Adsorbed on Porous Carbons. *J. Phys. Chem. C* **2014**, *118*, 7508-7514.

(10) Lyu, D.; Märker, K.; Zhou, Y.; Zhao, E. W.; Gunnarsdóttir, A. B.; Niblett, S. P.; Forse, A. C.; Grey, C. P. Understanding Sorption of Aqueous Electrolytes in Porous Carbon by NMR Spectroscopy. *J. Am. Chem. Soc.* **2024**, *146*, 9897-9910.
